# A fuzzy rough copula Bayesian network model for solving complex hospital service quality assessment

**DOI:** 10.1007/s40747-023-01002-w

**Published:** 2023-03-24

**Authors:** He Li, Mohammad Yazdi, Hong-Zhong Huang, Cheng-Geng Huang, Weiwen Peng, Arman Nedjati, Kehinde A. Adesina

**Affiliations:** 1 School of Intelligent Systems Engineering, Sun Yat-Sen University, Shenzhen, 518107 People’s Republic of China; 2 Faculty of Engineering and Applied Science, Memorial University of Newfoundland, St. John’s, NL A1B 3X5 Canada; 3 Center for System Reliability and Safety, University of Electronic Science and Technology of China, Chengdu, 611731 Sichuan People’s Republic of China; 4 Industrial Engineering Department, Quchan University of Technology, Quchan, Iran; 5 Industrial Engineering Department, Near East University, KKTC, Nicosia, North Cyprus Turkey

**Keywords:** MCDM, Bayesian analysis, Fuzzy set theory, Hospital service quality, Operation management

## Abstract

Healthcare tends to be one of the most complicated sectors, and hospitals exist at the core of healthcare activities. One of the most significant elements in hospitals is service quality level. Moreover, the dependency between factors, dynamic features, as well as objective and subjective uncertainties involved endure challenges to modern decision-making problems. Thus, in this paper, a decision-making approach is developed for hospital service quality assessment, using a Bayesian copula network based on a fuzzy rough set within neighborhood operators as a basis of that to deal with dynamic features as well as objective uncertainties. In the copula Bayesian network model, the Bayesian Network is utilized to illustrate the interrelationships between different factors graphically, while Copula is engaged in obtaining the joint probability distribution. Fuzzy rough set theory within neighborhood operators is employed for the subjective treatment of evidence from decision makers. The efficiency and practicality of the designed method are validated by an analysis of real hospital service quality in Iran. A novel framework for ranking a group of alternatives with consideration of different criteria is proposed by the combination of the Copula Bayesian Network and the extended fuzzy rough set technique. The subjective uncertainty of decision makers’ opinions is dealt with in a novel extension of fuzzy Rough set theory. The results highlighted that the proposed method has merits in reducing uncertainty and assessing the dependency between factors of complicated decision-making problems.

## Introduction

Hospital quality service assessment is critical for hospital management. The modern lifestyle of society requires extensive satisfaction with the quality and efficiency of hospital services. During the past year of the pandemic, healthcare and hospitals have proved to be one of the world's most highly complicated and significant sectors. The main aspects of hospital service quality include (not limited to) equipment, staff behavior, admitting, and several more directly related to patients, for instance, payment and treatment time [1]. Patient satisfaction is a degree of matching between the services that patients receive from the hospital and their expectations [2]. Therefore, improving the service quality and efficiency of hospitals’ services is a demanding task for decision makers and managers.

To assess the quality of hospital services, health care systems, and similar application domains, multi-criteria decision-making (MCDM) is typically utilized; see in [3] and [4]. In MCDM tools, a set of alternatives are examined simultaneously with consideration of different criteria. Techniques to address MCDM methods can be classified into four categories: (i) measurement tools (i.e., allocating a score for all the alternatives such as analytical hierarchy process (AHP) [5], and Evidence theory [6]); (ii) reference level methods (i.e., using an aggregation function such as TOPSIS (Technique for Order Preference by Similarity to an Ideal Solution) [7, 8], and VIKOR (Multicriteria Optimization and Compromise Solution) [9, 10]; (iii) outranking methods (i.e., comparing the pairwise comparisons for all single criterion such as PROMETHEE (king Organization Method for Enrichment Evaluation) [11], ELECTRE I, II, III, IV (Elimination and Choice Expressing Reality) [12], and QUALIFLEX (qualitative flexible multiple criteria method) [13]); (iv) other methods. MCDM tools are capable of handling complicated decision-making problems and are applicable in many application domains, including, but not limited to, business [14], process [15, 16], safety [17, 18], and supply chain [19]. More relevant, the BWM [20] has been maturely utilized in hospital service quality assessment [21].

In this study, the Copula Bayesian Network is employed to evaluate the service quality of a hospital. The decision makers' subjective uncertainty is dealt with in a novel extension of fuzzy rough set theory, and an integrated ranking system is presented to prioritize the alternatives. The study aims to design a structure to evaluate the service quality of hospitals. The outcomes could help the managers and decision makers to systematically prioritize the factors and spend the budget in a way that effectively improves the service quality of hospitals.

The rest of the paper is organized as the following. Related literature is presented in “Methodology”. Methodologies for analyzing hospital service quality, including the Bayesian Network, Copula functions, and the extended fuzzy rough set theory, are provided in “Application of study”. “Conclusion” demonstrates the application of the study provided to assess and evaluate a hospital service quality. Finally, conclusions and future discussions are listed in the last section.

### Related literature

Researchers applied various MCDM methods and different integrations of techniques to evaluate the healthcare service quality in recent years due to its significance and the presence of too many qualitative and quantitative factors. However, service quality is vital for the survival of any service-based company; hospitals and healthcare institutes are at the core of the concentration. In the first study [1], the authors used MCDM methods to evaluate the service quality of B-class hospitals in Istanbul. They used AHP to find the importance weight of criteria, then TOPSIS and Yager’s min–max approach were applied to rank the crisp performance values, and finally, OWA and Compensatory AND operators were employed to aggregate the result. In another study [2], a group of scholars used MCDM tools to identify and evaluate criteria influencing public hospitals in Iran. They used four hybrid methods and integrated the results by the Copeland method to achieve the main criteria of environment, responsiveness, equipment, facilities, and professional capability. Another study [3] employed a belief function theory to improve the BWM method as a framework to assess the hospital service quality problem. They tried to tackle the vagueness of decision makers in qualitative judgment through these integrations. The evaluation based on the patient's view is also investigated by [4] in a real case study in Istanbul. They used the Interval Valued Intuitionistic Fuzzy concept to improve TOPSIS to cope with the vagueness and complexity of evaluation. Another study integrated the fuzzy sets theory and the VIKOR method to evaluate hospital service quality in Taiwan [5]. They addressed vagueness, subjectivity, and uncertainty with linguistic variables in triangular fuzzy number format.

In Croatia [6], AHP is used to measure the quality of public hospitals. They ranked the top-performing hospitals in the country. According to the study of [7], an integrated distance-based Pythagorean Fuzzy method, TOPSIS, and Fuzzy Inference System design a framework that could evaluate the healthcare service quality of hospitals. Their approach is applied to a real case study for prioritizing the ten clinics in a private hospital. Pythagorean Fuzzy TOPSIS is used to determine the inputs of the fuzzy system, and the fuzzy inference system is applied to evaluate the clinic's service quality level. A study of [9] investigated the service performance evaluation of hospitals in the recent COVID-19 situations not only for health services but also for the elimination of hesitations in the treatment and vaccination processes. They integrated CRITIC-TOPSIS with fuzzy sets and designed a framework to evaluate the hospitals, and they suggested the required policies and strategies for hospitals under pandemic situations. Interested readers could also refer to [4] for more comprehensive information about MCDM and healthcare service quality evaluation. However, MCDM methods still suffer from a couple of shortages [22, 23]: (i) subjective input information causes subjective uncertainty of results; (ii) insufficient consideration of correlations between factors; (iii) disability in diagnosis analysis; (iv) insufficient in dealing with stochastic-based decision-making problems. Bayesian Network is an asset for model and analyzing the dependence of systems and is proved to be a helpful tool in several fields, such as safety and risk analysis [24–26], human reliability analysis [27, 28], resilience analysis [29], marine engineering [30, 31], and others. Bayesian Network is constructed according to the Bayesian inference process that can update the Bayesian Network with both predictive and diagnostic analysis once new evidence(s) are obtained. Table 1 shows the related publications that mostly applied Fuzzy-AHP and Fuzzy-TOPSIS integrations, and Bayesian network was somehow neglected in hospital service evaluation problem studies.

**Table 1:** Related literature approaches

Study	Problem	Approach
An integrated evaluation model for service quality of hospitals: a case study from Turkey [11]	Rank the hospitals according to service quality from the patient's point of view	Fuzzy-AHP and fuzzy TOPSIS
A fuzzy framework to evaluate service quality in the healthcare industry: an empirical case of public hospital service evaluation in Sicily [12]	Evaluate service quality in the public healthcare sector	Fuzzy-AHP
Evaluating the service quality of the hospital using TOPSIS with interval type-2 fuzzy sets [13]	Analyze and evaluate the service quality performance of the hospital in Taiwan	Fuzzy TOPSIS
A combined fuzzy multi-criteria decision-making approach for evaluating hospital website quality [14]	Measuring the website quality of hospitals	Fuzzy-DEMATEL
Hospital service quality evaluation: an integrated model based on Pythagorean fuzzy AHP and fuzzy TOPSIS [15]	Private hospital service quality evaluation	Pythagorean fuzzy AHP and fuzzy TOPSIS
Estimating the effect of health service delivery interventions on patient length of stay: a Bayesian survival analysis approach [17]	Estimating the effect of service delivery interventions on patient length of stay	Bayesian structural survival model

Moreover, the probability distributions can be engaged to tackle the objective uncertainties by describing the continuous variables in Bayesian Network. Bayesian Network also has considerable capability to inconsistent aggregate information, quantify different uncertainties, measure dependency between the factors, and have high flexibility and efficiency to make optimum decision-making [32, 33]. Accordingly, Bayesian Network and its extensions can be utilized to address the drawbacks of MCDM tools by constructing the Bayesian Network according to the prior knowledge that comes up from decision-makers' opinions or learning the Network using conditional probability based on the considerable input data.

Bayesian Network can be utilized to make a marginal decision to evaluate hospital service quality with consideration of confidence level. However, until now, no similar study to assess hospital service quality using Bayesian Network has been published. The typical Bayesian Network is still suffering from a couple of shortages when it is implemented in hospital quality service assessment, to be specific, including modeling marginal distributions and considering the dependency of interrelationships between factors based on the stochastic nature of the problem [34–36]. Another lack is that in the typical Bayesian Network, the conditional probability tables will be larger and larger by increasing the number of variables, making the problem too complex to solve. Copula Bayesian Network is developed to address the complicated dependencies of continuous variables using marginal distributions and dependency functions to deal with this issue. In addition, the Copula Bayesian Network can adequately model the dependence and causalities between variables, which further addresses the stochastic nature of decision-making problems [37, 38].

Considering the merits of the Copula Bayesian Network to solve a decision-making problem, subjective decision makers opinions still play vital roles in acquiring an important weight of criteria in hospital quality service. Therefore, in this study, an extension of fuzzy Rough set theory is used to cope with the ambiguities and uncertainty of subjective knowledge collected from decision makers which require no prior knowledge of decision makers and can also objectively handle the decision-making problems [39].

The contributions of this study are:

- The Copula Bayesian Network is used to analyze the hospital service quality.
- A novel extension of fuzzy Rough set theory based on neighborhood operators is engaged to deal with the subjective uncertainty of decision-makers' opinions.
- A framework for ranking a group of alternatives considering different criteria is proposed by combining the Copula Bayesian Network and the extended fuzzy Rough set technique.

## Methodology

**Fig. 1:**
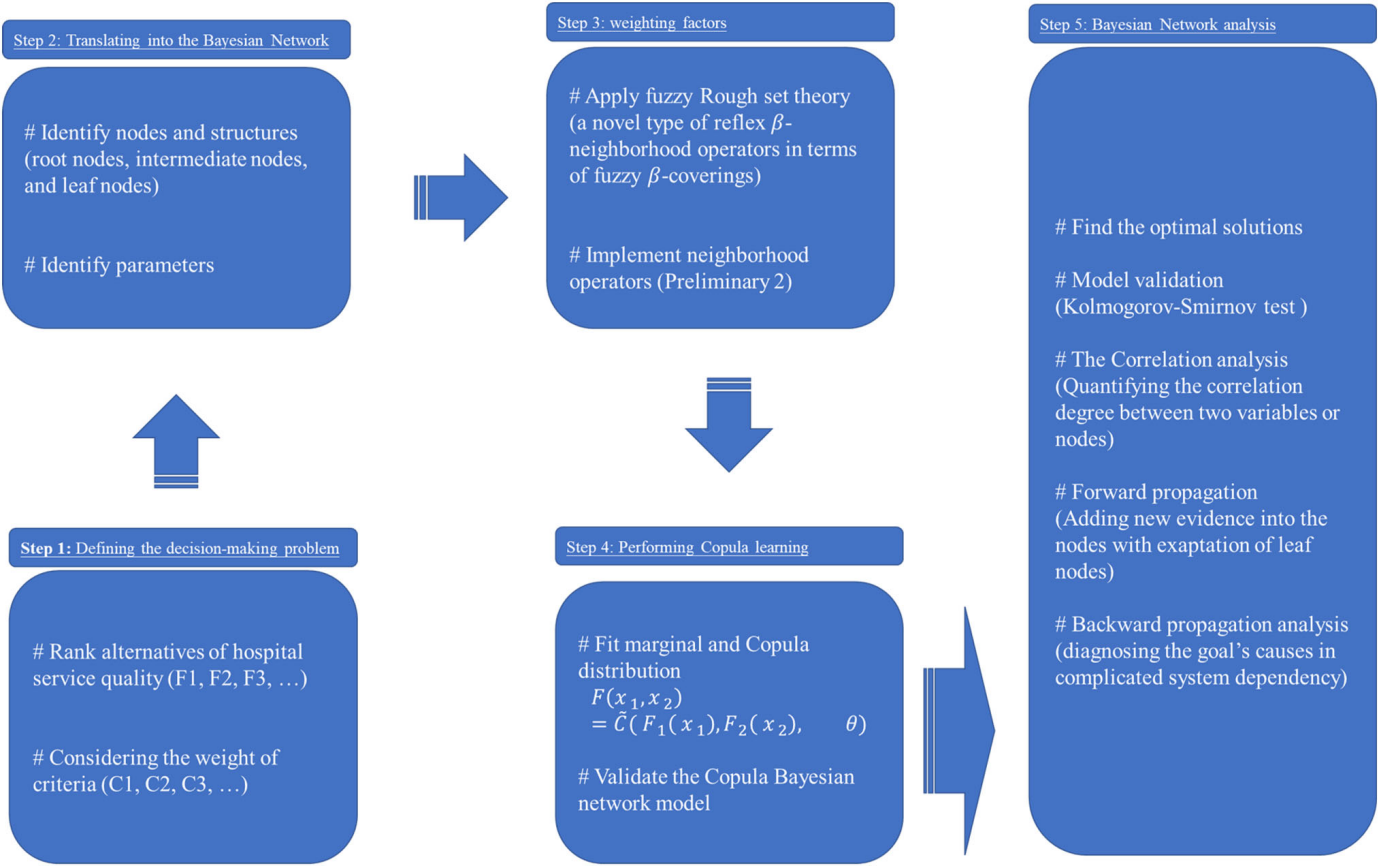
The proposed framework to obtain the optimum solutions

In this section, a five-step-based methodology is proposed to solve a decision-making problem by finding the optimum solutions, see Fig. 1.

### Step one: defining the decision-making problem

Decision-making problems in an ordinary way fall into a problem to solve or a decision to make. The initial schedule of solving the mentioned task is to define the problem (i.e., finding the highest priority for an alternative among a set of alternatives). In this study, the decision-making problem is designed to rank the alternatives in descending order by considering the importance weight of the criteria. Accordingly, first, all potential alternatives, together with criteria, should be recognized.

### Step two: translating the decision-making problem into a Bayesian Network model

Bayesian Network is a well-known probabilistic tool for constructing conditional dependency among a set of variables. The translation of the decision-making problem into a Bayesian Network model is to structure, in detail, the decisive goal and criteria (see Step 1). To be specific, Bayesian Network is a well-known probabilistic tool to construct conditional dependency among a set of variables consisting of two parts: directed acyclic graph and Conditional Probability Tables (CPTs). In the directed acyclic graph, nodes are variables, and edges are causalities between nodes. Nodes can be categorized into root nodes (no edge points to), intermediate nodes (with both starts-with and point-to edges), and leaf nodes (without start-with edges).

Bayesian Network propagates probabilistic information by the conditional probability function [40], as:

1 $$ P\left( {A{|}B} \right) = \frac{{P\left( {B{|} A} \right) \times P\left( A \right)}}{P\left( B \right)}, $$

where $P{(}A{|}B)$ represents the probability of node A given the state of node B, $P\left( A \right)$ and $P\left( B \right)$ denote prior probabilities of nodes $A$ and $B$

Assume that in a typical BN, $n$ variables as $A_{ 1} , A_{ 2} , A_{ 3} , \ldots , A_{ n}$, are included. In this accordance, the joint probability distribution of variables is decomposed as:

2 $$\begin{aligned} P\left( { A_{ 1} ,A_{ 2} , A_{ 3} , \ldots ,A_{ n} } \right) &= P\left( { A_{ 1} |A_{ 2} ,A_{3} , \ldots ,A_{ n} } \right)\\& \,\,\,\,\,\times P\left( {A_{2} |A_{3} , \ldots ,A_{n} } \right) \ldots\\& \,\,\,\,\,\times P\left( {A_{n - 1} |A_{ n} } \right). \end{aligned}$$

Equation (2) can be simplified into Eq. (3) according to the D-separation rule [20], as:

3 $$ \begin{aligned} P\left( {A_{ 1} ,A_{2} ,A_{3} , \ldots ,A_{n} } \right)& = \mathop \prod \limits_{i = 1}^{ n } P\left( {A_{i} | A_{i + 1} ,A_{i + 2} , \ldots ,A_{n} } \right) \\&= \mathop \prod \limits_{i = 1}^{ n } P\left( {A_{i} |{\text{Parrents}}\left( {A_{i} } \right)} \right).\end{aligned} $$

Assume that a typical BN is structured having a set of limited variables as $M = \left\{ {A_{ 1} ,A_{ 2} ,A_{ 3} ,A_{ 4} } \right\}$, and consists of a set of arcs that illustrates the interdependency and relationships between the existing variables. Bayesian Network is a robust and powerful decision-making tool compared to the other existing analysis method. The reason for utilizing BN in this paper are highlighted as the following: (i) graphical representation as a network helps decision makers that could track the process and have a better undersetting of the problem, (ii) BN could engage both objective and subjective data as an input, (iii) it also could handle the uncertainty as well as updating the information.

Up to this point, Bayesian Network is briefly explained. Next, Decision-makers should identify all factors and subfactors related to the alternative selection. Afterwards, the importance weight of all factors and corresponding subfactors should be computed, which will further be explained in step three. Finally, the causality between the factors and subfactors will be determined to better understand the cause-and-effect relationship within the factors.

### Step three: collecting experts’ opinions for factor weighting

This step gathers a heterogeneous group of decision makers (e.g., four to six individuals). It should be added that all of the called decision makers must have a relevant background, expertise, or education regarding the application of study and have a proper understanding of the proposed methodology, the idea behind the work, and how their contributions are essential and can considerably value to the scientific communities. In addition, the group of decision makers should declare that there is not any conflict of interest as well as any kind of relationship that might impact the outcome of the elicitation process and the outcome of the investigations. A group of experts as decision makers should be employed to provide a proper significant weight for the factors involved. In this study, an extension of the fuzzy Rough set theory is improved to collect and aggregate decision-makers’ opinions. The aggregation of experts' opinions into BN is extensively discussed in previous authors' published work. To avoid repetition and duplication, an interested reader can refer to the following references [41, 42].

Dubios and Prade [37] propose the fuzzy Rough set theory[43]. Afterwards, several extensions have been proposed to meet actual engineering requirements, and the most popular ones are fuzzy Rough set models [44, 45]. Such studies extended the technique by replacing the fuzzy binary relations with a fuzzy covering or replacing the fuzzy binary relations with fuzzy neighborhood operators. However, the fuzzy covering is strict, which raises difficulties in common decision-making problems.

To deal with this situation, the concept of $\beta$-coverings is proposed in [46], in which two different fuzzy Rough sets are presented by defining a fuzzy $\beta$-neighborhood. Accordingly, Yang and Hu [47] worked to expand the theoretical knowledge related to fuzzy $\beta$-coverings estimation space underlying the idea of fuzzy Rough set theory and fuzz $\beta$-neighborhood operators. It should be noted that the four types of fuzzy $\beta$-neighborhood operators proposed in [47] can be further extended to the four categories of fuzzy $\beta$-neighborhood operators [48]. However, the fuzzy $\beta$-neighborhood operators cannot satisfy the relexification feature, which is also a considerable shortage of the fuzzy Rough set theory. Therefore, in this study, we unutilized a novel type of reflex $\beta$-neighborhood operators in fuzzy $\beta$-coverings [49].

The proposed $\beta$-neighborhood operators are defined in the following subsection.

#### Theories and definitions

Some of the main fuzzy operators can be summarized as the following [39]:

(I) There are three types of $t$-norms (showing as$\tau$) for all $c ,d\in \left[0 ,1\right] ,$
• The standard minimum operator:${\tau }_{ M }\left(c ,d\right)=\mathrm{min}(c ,d)$,
• The algebraic product: ${\tau }_{P }\left(c ,d\right)=c*d$,
• The Lukasiewics $${\tau }_{ L }\left(c,d\right)=\mathrm{max}( 0 ,c+d-1)$$,
(II) There are three types of R-implicators (showing as $\Lambda )$ for all $c ,d\in \left[\mathrm{0,1}\right] ,$
• It is Godel implicator according to the $\tau_{ M}$
  $$ {\Lambda }_{{ \tau_{ L } }} \left( {c ,d} \right) = \left\{ {\begin{array}{*{20}c} {1 c \le d} \\ {d d > c} \\ \end{array} ,} \right. $$
  where R-implicators for all$c ,d \in \left[ { 0, 1} \right] , \tau_{ L } \left( {c ,d} \right) = {\text{sup}}\{ c \in \left[ {0 , 1} \right)| \tau_{ } \left( {c,d} \right) \le d$].
  It is Godel implicator according to the $\tau_{ P}$
  $$ {\Lambda }_{{ \tau_{ L } }} \left( { c ,d} \right) = \left\{ {\begin{array}{*{20}c} {1 c \le d} \\ {d/c d > c} \\ \end{array} } \right. $$
• It is Lukasiewics implicatory according to the ${\tau }_{ L }:{\Lambda }_{{ \tau }_{ L }}\left( c ,d\right)=\mathrm{min}(c ,c-c+d)$

According to the fuzzy neighborhood operators, some of the fuzzy covering can be summarized as follows.

##### Definition 1

[46]. Let us assume that $U$ is a universal set where $F\left( U \right)$ denotes a fuzzy family set of $U$. Assume that $\tilde{\delta } = \left( {\delta_{ 1} ,\delta_{ 2} , \ldots ,\delta_{ m} } \right)$ with $\delta_{ j} \in F\left( U \right)$ and $C = \left( {1,2, \ldots m} \right)$ would be an index for all $k \in C$. For each $\beta \in \left[ { 0,1} \right]$, $\tilde{\delta }$ is named a fuzzy $\beta$-covering of $U$ satisfying $\left( {\bigcup\nolimits_{j = 1}^{m} {\delta_{m} } } \right)\left( {a \ge \beta } \right)$ for all $a \in U$. Then, the pair of $\left( {U,\tilde{\delta }} \right)$ is named a fuzzy $\beta$-covering estimation space and illustrated as F $\beta CAS$

##### Definition 2

Let us assume that $\left( {U,\tilde{\delta }} \right)$ is a F $\beta CAS$ for some $\beta \in \left( {0 ,1} \right]$ and$\tilde{\delta } = \left( {\delta_{ 1} ,\delta_{ 2} , \ldots ,\delta_{ m} } \right)$. Then for every$a \in U$, the $\beta$-neighborhood system can be written as:

4 $$ {\Delta }_{ \beta }^{{ \tilde{\delta }}} = \{ \delta_{ j} \in \tilde{\delta }|\delta_{ j} \left( a \right) \ge \beta \} . $$

In addition, the fuzzy $\beta$-neighborhood operator can be defined as the following:

Where ${\Delta }_{ \beta , Ma }^{{ \tilde{\delta }}} \left( a \right)\left( a \right) \ge \beta$ for each $a \in U$.

The operators ${\Delta }_{ \beta , Ma }^{{ \tilde{\delta }}}$ is somehow $\beta$-reflexive, and therefore this operator cannot satisfy the reflexivity when $\beta \ne 1$.

5 $$\begin{aligned} {\Delta }_{ \beta ,Ma }^{{ \tilde{\delta }}}& = \{\delta_{ j} \left( b \right){|}\delta_{ j} \in \tilde{\delta }| \delta_{ j} \left( a \right) \ge \beta \} .\end{aligned} $$

##### Definition 3

[47]**.** Let us assume that $\left( {U,\tilde{\delta }} \right)$ is a F $\beta CAS$ for some value of $\beta \in \left( {0 ,1} \right]$ and$a \in U$, the fuzzy $\beta$-minimal description $\widetilde{Md}_{{\beta , \tilde{\delta }}}^{{{\text{ Yang }}\& {\text{ Hu}} }} \left( a \right)$ and fuzzy $\beta$-maximal description $\widetilde{Md}_{{ \beta , \tilde{\delta }}}^{{{\text{ Yang }}\& {\text{ Hu}} }} \left( a \right)$ can be defined as the following:

6 $$ \widetilde{Md}_{{ \beta ,\tilde{\delta }}}^{{{\text{Yang }}\& {\text{Hu}} }} \left( a \right){ } = \{ \delta_{ j} \in {\Delta }_{ \beta }^{{ \tilde{\delta }}} |\left( {\forall \delta_{j} \in {\Delta }_{ \beta }^{{\widetilde{ \delta }}} \left( a \right)} \right)\left( {\delta_{ i} \subseteq \delta_{ j} \Rightarrow \delta_{ i} = \delta_{ j} } \right) $$

7 $$ \widetilde{Md}_{{ \beta , \tilde{\delta }}}^{{{\text{Yang }}\& {\text{Hu}} }} \left( a \right){ } = \{ \delta_{ j} \in {\Delta }_{ \beta }^{{\widetilde{ \delta }}} |\left( {\forall \delta_{j} \in {\Delta }_{ \beta }^{{\widetilde{ \delta }}} \left( a \right)} \right)\left( {\delta_{ i} \supseteq \delta_{ j} \Rightarrow \delta_{ i} = \delta_{ j} } \right) $$

##### Definition 4

Let us assume that $\psi \left( {\delta ,a} \right)$ denotes a fuzzy neighborhood system of $a$ when$a \in U$, in which $\psi \left( {\delta ,a} \right) = \left\{ {\delta_{ j} \in \delta \left| { \delta_{ j} \left( a \right)} \right\rangle 0} \right\}$. Accordingly, the fuzzy minimal and maximal descriptions of $a$ as $\widetilde{Md}\left( {\delta ,a} \right)$ and$\widetilde{MD}\left( {\delta ,a} \right)$, respectively, can be presented as the following:

8 $$ \widetilde{Md}\left( {\delta ,a} \right){ } = \{ \delta_{ j} \in \left( {\delta ,a} \right)|\left( {\forall \delta_{ j} \in \left( {\delta ,a} \right)} \right)\left( {\delta_{ i} \left( a \right) = \delta_{ j} \left( a \right) , \delta_{ i} \subseteq \delta_{ j} \Rightarrow \delta_{ i} = \delta_{ j} } \right), $$

9 $$ \widetilde{MD}\left( {\delta ,a} \right){ } = \{ \delta_{ j} \in \left( {\delta ,a} \right)|\left( {\forall \delta_{ j} \in \left( {\delta ,a} \right)} \right)\left( {\delta_{ i} \left( a \right) = \delta_{ j} \left( a \right) , \delta_{ i} \supseteq \delta_{ j} \Rightarrow \delta_{ i} = \delta_{ j} } \right). $$

According to the mentioned equations, four fuzzy neighborhood operators were proposed by D’eer et al. [50]. Let us assume that $\left( {U,\tilde{\delta }} \right)$ is a finite fuzzy covering estimation space (FCAS), $\tau$ a $t$-norm and $L$ an implication. Therefore, for $\forall a,b \in U$, the following operators can be defined:

10 $$ {\Delta }_{ 1 }^{{\widetilde{ \delta }}} \left( a \right)\left( b \right) = {\text{inf}}_{{ \delta_{ i} \in \delta }} L\left( {\delta_{ i} \left( a \right),\delta_{ i} \left( b \right)} \right), $$

11 $$ {\Delta }_{ 2 }^{{ \widetilde{\delta }}} \left( a \right)\left( b \right) = {\text{sup}}_{{ \delta _{i} \in \widetilde{ Md}\left( {\delta ,a} \right)}} \tau \left( {\delta_{ i} \left( a \right),\delta_{ i} \left( b \right)} \right), $$

12 $$ {\Delta }_{ 3 }^{{ \tilde{\delta }}} \left( a \right)\left( b \right) = {\text{inf}}_{{\delta_{ i} \in \widetilde{MD}\left( {\delta , a} \right)}} L\left( {\delta_{ i} \left( a \right),\delta_{ i} \left( b \right)} \right), $$

13 $$ {\Delta }_{ 1 }^{{ \tilde{\delta }}} \left( a \right)\left( b \right) = {\text{sup}}_{{\delta_{ i} \in \delta }} \tau \left( {\delta_{ i} \left( a \right),\delta_{ i} \left( b \right)} \right), $$

where the fuzzy covering $\delta$ is a crisp covering, and the four mentioned fuzzy neighborhood operators are fully reflexive. The first and third operators are properly transitive, and the last operator is symmetric.

##### Remark 1

Intuitionistic fuzzy numbers are one of the main essential types of fuzzy numbers and are widely used in fuzzy operators. To obtain more information related to the intuitionistic fuzzy number, one can refer to [51, 52].

Recently, four novel types of fuzzy $\beta$-neighborhood operators have been proposed by Ye et al. [49] to deal with the shortcoming of existing neighborhood operators such as [47]. In the following, these three novel operators in a finite F $\beta$ CAS are explained.

##### Definition 5

Let us assume that $\left( {U,\tilde{\delta }} \right)$ is a finite F $\beta$ CAS, $\tau$ is a $t$-norm and $L$ is an implicator, for $a ,b \in U$, the operators ${\Delta }_{{ \widetilde{\delta },s }}^{ \beta }$ ($s = 1, 2, 3, {\text{and}} \, 4):U = \acute{F} \left( U \right):a \to {\Delta }_{{\tilde{\delta },s }}^{ \beta } \left( a \right)$ are redefined the four types mentioned above of fuzzy $\beta$-neighborhood operators. ${\Delta }_{ \delta ,s }^{ \beta }$ ($s = 1,2,3,{\text{ and }}4)$ can be therefore defined as the following order:

14 $$ {\Delta }_{{\widetilde{ \delta }, 1 }}^{ \beta } \left( a \right)\left( b \right) = {\text{inf}}_{{\delta_{ i} \in \delta }} L (\delta_{ i} \left( a \right),\delta_{ i} \left( b \right)), $$

15 $$ {\Delta }_{{ \tilde{\delta },1 }}^{ \beta } \left( a \right)\left( b \right) = {\text{sup}}_{{ \delta_{i } \in \widetilde{Md}\left( {\delta , a} \right)}} \tau \left( {\delta_{ i} \left( a \right),\delta_{ i} \left( b \right)} \right), $$

16 $$ {\Delta }_{{ \tilde{\delta },1 }}^{ \beta } \left( a \right)\left( b \right) = {\text{inf}}_{{\delta_{ i} \in \widetilde{MD}\left( {\delta , a} \right)}} L(\delta_{ i} \left( a \right),\delta_{ i} \left( b \right)), $$

17 $$ {\Delta }_{{ \tilde{\delta },1 }}^{ \beta } \left( a \right)\left( b \right) = {\text{sup}}_{{ \delta_{i } \in \delta }} \tau \left( {\delta_{ i} \left( a \right),\delta_{ i} \left( b \right)} \right). $$

According to Eqs. (14)–(17), the following results can be concluded:

- If $\tilde{\delta }$ is a fuzzy covering, Eqs. (14)–(17) can degenerate into Eqs. (6)–(9), respectively. They would be called as ${\Delta }_{{\widetilde{ \delta },1 }}^{ 1} = {\Delta }_{1}^{{\tilde{\delta }}} ,{\Delta }_{{ \tilde{\delta },2 }}^{ 2} = {\Delta }_{2 }^{{\widetilde{ \delta }}}$,${\Delta }_{{\widetilde{\delta }, 3 }}^{ 3} = {\Delta }_{3 }^{{\tilde{\delta }}}$, and${\Delta }_{{ \tilde{\delta }, 4 }}^{ 4} = {\Delta }_{ 4 }^{{\widetilde{ \delta }}}$.
- If $\tilde{\delta }$ is a fuzzy covering, then ${\Delta }_{{\widetilde{ \delta }, 1 }}^{ 1} \left( a \right) \subseteq {\Delta }_{{\widetilde{ \delta }, 1 }}^{ 1,Ma}$(a),${\Delta }_{{\widetilde{ \delta }, 2 }}^{ 2, Ma } \left( a \right) \subseteq {\Delta }_{{\widetilde{ \delta }, 2 }}^{ 1} \left( a \right)$, $\Delta_{{\widetilde{\delta },3}}^{ 1} \left( a \right) \subseteq \Delta_{{\widetilde{\delta },3}}^{3,Ma}$(a), and${\Delta }_{{\widetilde{ \delta }, 1 }}^{{ 4, {\text{Ma}} }} \left( a \right) \subseteq {\Delta }_{{\widetilde{ \delta }, 4 }}^{ 1} \left( a \right)$.

If $\tilde{\delta }$ is a crisp covering, the operators ${\Delta }_{{\widetilde{ \delta }, s }}^{ \beta }$ ($s = 1, 2, 3, {\text{and}} \, 4)$ overlap with four conventional kinds of neighborhood operators, which are defined by Yao et al. [49].

It should be highlighted that the computation of operators ${\Delta }_{{\widetilde{ \delta }, 4 }}^{ \beta }$ and ${\Delta }_{{\widetilde{ \delta }, 1 }}^{ \beta }$ are independent of the factor$\beta$. Once all opinions are collected from decision makers in any form of fuzzy numbers, all the fuzzy numbers can be aggregated into a crisp number by implementing the above methodology.

### Step four: performing Copula learning

A Copula is a function to create a joint multivariate distribution in which one dimension of marginal distribution would be combined. Copula has enough capability, such as having considerable flexibility in structural characterizing. Moreover, it is a robust and powerful tool for selecting a probability distribution, even in mistaken selection [53]. Besides, the $n$-dimensional continues multivariate random numbers as vector $x = \left( {x_{ 1} ,x_{ 2} , \ldots x_{ n} } \right)$ has this chance to be reformed based on $n$ univariate marginal distributions $F_{ 1} \left( {x_{ 1} } \right),F_{ 2} (x_{ 2} ), \ldots ,F_{ n} (x_{ n} )$ and $n$-dimensional Copula function $\tilde{C}$, which is defined in the following equations. The Copula function $\tilde{C}\left[ {0,1} \right]^{ d} \to \left[ {0,1} \right]$ maps univariate the marginal joint cumulative distributions $F_{ 1} \left( { x_{ 1} } \right),F_{ 2} ( x_{ 2} ), \ldots ,F_{ n} ( x_{ n} )$ into the joint distribution $F$ [54].

18 $$ F\left( { x_{ 1} ,x_{ 2} , \ldots x_{ n } } \right) = \tilde{C}\left( { F_{ 1} \left( {x_{ 1} } \right),F_{ 2} (x_{ 2} } \right), \ldots ,F_{ n} (x_{ n} ){ }). $$

Also, when the marginals are continuous, $\tilde{C}$ can be explained by:

19 $$ \tilde{C} \left( { u_{ 1} ,u_{ 2} , \ldots u_{ k} } \right) = F\left( { F _{1}^{ - 1} \left( {u_{ 1} } \right), F_{ 1}^{ - 1} \left( {u_{ 2} } \right), \ldots , F_{ n}^{ - 1} \left( { u_{ n} } \right)} \right), $$

where $F_{ i} \left( {x_{ i} } \right)$ $\forall i \in \left\{ {1, 2, \ldots n} \right\}$ is the marginal distribution of $x_{ i}$, and $\tilde{C}$ is based on the Copula function, and $u_{i} = F_{ i} \left( { x_{ i} } \right)$ for $i \in \left\{ {1, 2, \ldots n} \right\}$. Moreover, for the bivariate distribution, $F\left( {x_{ 1} ,x_{ 2} } \right)$ can be shown in terms of the Copula function and two different marginal joint cumulative distributions as:

20 $$ F\left( {x_{ 1} ,x_{ 2} } \right) = \tilde{C}\left( { F_{ 1} \left( { x_{ 1} } \right),F_{ 2} \left( { x_{ 2} } \right), \theta } \right). $$

In which, $\theta$ is signified by the Copula parameter to calculate the dependency of two different variables $x_{ 1}$ and $x_{ 2}$, defined by the Pearson correlation coefficient and denoted as $\rho$. The parameter $\rho$, therefore, be obtained as:

21 $$\begin{aligned} \rho &= \mathop\int \limits_{- \infty }^{\infty }
\mathop \int \limits_{ - \infty }^{\infty } \left( {\frac{{ x_{
1 - } \mu_{{ x_{ 1} }} }}{{\sigma_{{ x_{ 1} }} }}} \right)\left(
{\frac{{ x_{{ 2 - \mu_{{ x_{ 2} }} }} }}{{\sigma_{{ x_{ 2} }} }}}
\right)f_{ 1} \left( {x_{ 1} } \right)f_{ 2} \left( {x_{ 2} }
\right) \partial \widetilde{C }\\ &\quad \times \left( {\frac{{F_{
1} \left( {x_{ 1} } \right), F_{ 2} \left( { x_{ 2} } \right),
\theta }}{{\partial F_{ 1} \left( {x_{ 1} } \right)\partial F_{ 2}
\left( {x_{ 2} } \right){\text{d}}x_{ 1} {\text{d}}x_{ 2} }}}
\right). \end{aligned}$$

In which, $\mu_{{x_{1} }}$ and $\mu_{{x_{2} }}$ are the mean values of $x_{ 1}$ and $x_{ 2}$, $\sigma_{{ x_{ 1} }}$ and $\sigma_{{ x_{ 2} }}$ reflect the standard deviation of $x_{ 1}$ and $x_{ 2}$, and $f_{ 1} \left( {x_{ 1} } \right)$ and $f_{ 2} \left( {x_{ 2} } \right)$ represent the marginal probability density function of $x_{1}$ and $x_{2}$, respectively.

Integrating Copula into Bayesian Network to create Copula Bayesian Network models supports the handling of complex decision-making problems, as it can fully consider the dependency within the variables in the Network based on an existing database. Considering data availability from objective data or elicitation process from decision makers, Copula can be appropriately determined by two different aspects: marginal distributions to fit the variables’ properties and Copula functions to model dependency structure. The way of determining marginal distributions and Copula functions is provided as follows:

(i) Determining marginal distributions

The most significant task to evaluate the best-fitted marginal distribution for the variables is properly describing a probability distribution. Three types of marginal distributions are typically used Normal distribution, Beta distribution, and lognormal distribution, see Table 2. To evaluate the precision of marginal distributions, the comparison tools like the Akaike Information Criterion (AIC), see Eq. (18) is applicable. The AIC with minimum value shows that the best marginal distribution is fitted.

22 $$ {\text{AIC}} = - 2 \times {\text{log}}({\text{max likelihood}}) + 2 \times \left( {\text{number of parameters}} \right), $$

where the likelihood is the maximum value for the model.

**Table 2:** Three types of marginal distributions

Name of distribution	PDF	Mean	Standard deviation
Normal	$\frac{1}{{\sigma \sqrt {2\pi } }}{\text{exp}}\left( { - \frac{{\left( {x - \mu } \right)^{ 2} }}{{2\sigma^{ 2} }}} \right)$	$\mu$	$\sigma$
Log-normal	$\frac{1}{{x\sigma \sqrt {2\pi } }}{\text{exp}}\left( { - \frac{{\left( {\log x - \mu } \right)^{ 2} }}{{2\sigma^{ 2} }}} \right)$	${\text{exp}}\left( {\mu + \frac{{\sigma^{2} }}{2}} \right)$	$\sqrt {\left( {\exp \left( {\sigma^{ 2} } \right) - 1} \right){\text{exp}}\left( {2\mu + \sigma^{ 2} } \right)}$
Beta	$\frac{{\left( {x - a} \right)^{\alpha - 1} \left( {b - x} \right)^{ \beta - 1} }}{{\beta \left( {\alpha ,\beta } \right)\left( {b - a} \right)^{ \alpha + \beta + 1} }}$	$\frac{\alpha }{\alpha + \beta }$	$\frac{\alpha \beta }{{\left( {\alpha + \beta } \right)^{ 2} \left( {\alpha + \beta + 1} \right)}}$

*PDF* Probability Density Function

(ii) Determining the Copula function

Copula functions have unique characteristics such as tail dependency, symmetry, etc. Therefore, these Copula functions can be utilized to fit the various models and make an appropriate effect on the output’s viability. Besides, the Gaussian normal Copula, which is one of the most important and common Copula based on elliptical Copula, is presented as:

23 $$ Co_{ \rho }^{ G} (x_{ 1} ,x_{ 2} , \ldots x_{ n} ) = \phi_{ \rho } (\phi^{ - 1} (x_{ 1} ),\phi^{ - 1} (x_{ 2} ), \ldots \phi^{ - 1} (x_{n} )). $$

The Gaussian normal Copula is an $n$-dimensional generalization, easy to structure dependencies with uncertainty, and efficient in modeling bivariate distribution with a lack of data [45]. Therefore, Gaussian normal Copula among existing ones is selected in this study. The density function of Gaussian normal Copula is presented as:

24 $$ Co_{ \rho }^{ G} \left( {x_{ 1} ,x_{ 2} , \ldots x_{ n} } \right) = \frac{{\partial Co_{ \rho }^{ G} \left( {x_{ 1} ,x_{ 2} , \ldots x_{ n} } \right)}}{{\partial x_{ 1} , \partial x_{ 2} , \ldots \partial x_{ n} }} = \left| \rho \right|^{{\left( { - \frac{1}{2}} \right)}} {\text{ exp}}\left( { - \frac{1}{2}\xi \left( {\rho^{ - 1} - I} \right)} \right). $$

Thus, the main difference between the Gaussian normal Copula and joint cumulative distribution function is that the variables in the Gaussian normal Copula follow different types of the marginal cumulative distribution function, which provide a better firing with a complex system.

In Eqs. (19) and (20), the $\rho$ is the $n$-order symmetric positive definite with the ${\text{diag}}\left( \rho \right) = 1$, $\phi_{ \rho }$ is a standard multivariate normal distribution with correlation matrix $\rho$, $\phi^{ - 1}$ denotes the inverse function standard univariate normal cumulative distribution function $\xi = (\phi^{ - 1} (x_{ 1} ),\phi^{ - 1} (x_{ 2 } ), \ldots \phi^{ - 1} (x_{ n } ))$, and $I$ represent the unit matrix. Assume that the dimension of $n$ is equal to 2, the following Equation can determine the bivariate normal Copula, as:

25 $$ Co_{{\rho_{ 12} }}^{ G} \left( {u ,\nu } \right) = \mathop \int \limits_{ - \infty }^{{\phi^{ - 1} \left( u \right)}} \mathop \int \limits_{ - \infty }^{{\phi^{ - 1} \left( \nu \right)}} \frac{1}{{2\pi \sqrt {1 - \rho^{ 2} } }}\exp \left( { - \frac{{s^{ 2} + t^{ 2} - 2\rho st}}{2}} \right){\text{d}}s{\text{d}}t, $$

where $\rho_{ 12}$ represents the correlation coefficient of the bivariate standard normal distributions.

### Step five: Bayesian network analysis

In this section, four types of analysis are introduced to show the proposed model can be effectively used in decision-making problems, including (i) model validation, (ii) correlation analysis, (iii) forward propagation analysis, and (iv) backward propagation analysis.

#### Model validation

The Kolmogorov–Smirnov test is performed to estimate the goodness of the obtained best-fitting marginal. The Kolmogorov–Smirnov test calculates the distance within the empirical distribution and approximates the distribution’s function, see Eq. (26). In the null hypothesis at a significant level of 0.5%, the data shape a unique distribution when $h = 0$ and $p$-value $> 0.05$.

26 $$ D = {\varvec{Sup}}_{ - \infty < x < \infty } \left| { F_{{\text{ exp}}} \left( x \right) - F_{{{\text{abs}}}} \left( x \right)} \right|. $$

Which $F_{abs}$ follows the empirical distributions according to the collected data. $F_{ exp} \left( x \right)$ follows the approximated distribution, and the supremum of the measurement distance is ${\varvec{Sup}}$.

Similarly, the empirical Copula depends on the given data engaged in examining if the Gaussian Copula makes for the best fitting of the data. Assume that $\left( {x_{ i} ,y_{ i} } \right)$ $\left( {i = 1,2, \ldots n} \right)$ are a sample from $\left( {X,Y} \right)$. The empirical distribution functions of $X {\text{and}} Y$ can be presented by $F_{ n} \left( x \right)$ and $G_{ n} \left( x \right)$, respectively. Accordingly, the empirical bivariate Copula is defined as:

27 $$ \widehat{{Co_{n} }}\left( {u,\nu } \right) = \frac{1}{n}\mathop \sum \limits_{i = 1}^{ n} I_{{\left[ {F_{n} \left( {x_{i} } \right) \le u} \right]}} I_{{\left[ {G_{n} \left( {x_{i} } \right) \le \nu } \right]}} ,u,\nu \in \left[ {0,1} \right], $$

where $I_{\left[ , \right]}$ denotes the indicative function, and $F_{ n} \left( {x_{ i} } \right) \le u,I_{{\left[ {F_{ n} \left( {x_{ i} } \right) \le u} \right]}} = 1$; otherwise, $I_{{\left[ {F_{n } \left( {x_{i } } \right) \le u} \right]}} = 0$.

In addition, the empirical Copula can be compared with other types of Copulas according to the computation of Euclidean distance as the following Equation:

28 $$ d^{2} = \mathop \sum \limits_{i = 1}^{ n } \left| {\widehat{{Co_{n} }}\left( {u_{i} ,\nu_{i} } \right) - \widehat{Co}\left( {u_{i} ,\nu_{i} } \right)} \right|^{2} , $$

where $u_{ i} = F_{ n } \left( {x_{i} } \right),\nu_{i} = G_{n} \left( {x_{i} } \right)$ $\left( {i = 1,2, \ldots n} \right)$, $\widehat{{Co_{ n} }}\left( {u_{ i} ,\nu_{ i} } \right)$ stands for the empirical Copula, and $\widehat{Co}\left( {u_{ i} ,\nu_{ i} } \right)$ is the best-fitted Copula.

#### The correlation analysis

The Correlation analysis is to quantify the correlation degree between two variables or nodes. The standard correlation coefficient measures the linear relation between two variables and does not consider the impact of other variables. However, it may be the effect of the un-controlled variable on these two variables, which causes misleading outputs. To deal with this challenge, one can use the partial correlation coefficient to evaluate relationships between the two variables under the influence of other variables in the Network. For instance, variable $z$ is related to two variables of $x$ and $y$, and the partial correlation analysis of the $x$ and $y$ can be computed according to standard correlation as presented in Eq. (29), in which the output is between zero and 1, meaning that zero shows that there is no linear relationship and 1 or − 1 denotes the highest or lowest linear relationships:

29 $$ \gamma_{xy,z} = \frac{{\gamma_{xy} - \gamma_{xz} \gamma_{yz} }}{{\sqrt {\left( {1 - \gamma_{xz}^{2} } \right)\left( {1 - \gamma_{yz}^{2} } \right)} }}, $$

where $\gamma_{ xz}$ denotes the correlation between two variables $x$ and $z$, $\gamma_{ yz}$ is the correlation between the variables $y$ and $z$, $\gamma_{ xy, z}$ is the correlation between $x$ and $y$; both are un-correlated with variable $z$.

In addition, the Spearman ranking correlation has similarities with the partial correlation coefficient. If the result is closer to 1 or − 1, the relationships would be more robust. In addition, Spearman’s ranking correlation is significantly dependent on the ranking of each variable rather than the existing data, see Eq. (30):

30 $$ r_{R} = 1 - \frac{{6\mathop \sum \nolimits_{i}^{n} d_{i}^{2} }}{{n\left( {n^{2} - 1} \right)}}, $$

where $d_{i} = {\text{rank}}\left( {x_{i} } \right) - {\text{rank}}\left( {y_{i} } \right)$ is the gap in the ranks according to the element $i$ is somehow the paired set of data $x$ and $y$, and $n$ denotes the amount of data from the two variables $x$ and $y$.

#### Forward propagation

Forward propagation analysis is adding new evidence into the nodes with the exaptation of leaf nodes, in this case, Bayesian Network could be renovated by forwarding propagation. The goals provided in the leaf nodes evaluate how appropriate locations would exist for lift installation. This can be predicated in Eq. (31) following an assumption, which causes are mutually independent:

31 $$ P\left( z \right) = \mathop \sum \limits_{ x} P(z| x_{1} ,x_{2} , \ldots ,x_{n} )P\left( {x_{1} ,x_{2} , \ldots ,x_{n} } \right) = \mathop \sum \limits_{x} \left[ {P\left( {z{|} x_{1} ,x_{2} , \ldots ,x_{n} } \right)\mathop \prod \limits_{i = 1}^{ n} P\left( {x_{i} } \right)} \right], $$

where ($x_{ 1} ,x_{ 2} , \ldots .,x_{ n}$) is a group of random variables denoted the causes, and $z$ is the main goal in the Bayesian Network.

According to the Equation above, the marginal distribution with the leaf node’s mean and variance will be altered. Therefore, the best location can be evaluated compared to the effect in the Bayesian Network according to the different forward reasonings.

#### Backward propagation analysis

Backward propagation analysis is to diagnose the goal’s causes in complicated system dependency. Bayes' theorem is used to compute the posterior probability distribution of causes $x _{i}$. The distribution variation shows how much a cause can contribute to the consequence, see Eq. (32). In general, the greater is the change, the more significant the cause is in the location determination of the system:

32 $$ P\left( {x_{ i} {|}z} \right) = \frac{{P\left( { z{|} x_{ i} } \right)P\left( {x_{ i} } \right)}}{P\left( z \right)} = \frac{{P\left( { z{|} x_{ i} } \right) P\left( {x_{ i} } \right)}}{{\mathop \sum \nolimits_{x} \left[ { P\left( { z{|} x_{1} ,x_{2} , \ldots ,x_{n} } \right)\mathop \prod \nolimits_{i = 1}^{ n} P\left( {x_{i} } \right)} \right]}}, $$

where $P\left( {x_{ i} {|}z} \right)$ is the conditional probability for variable $x_{ i}$ given evidence $z$.

## Application of study

**Fig. 2:**
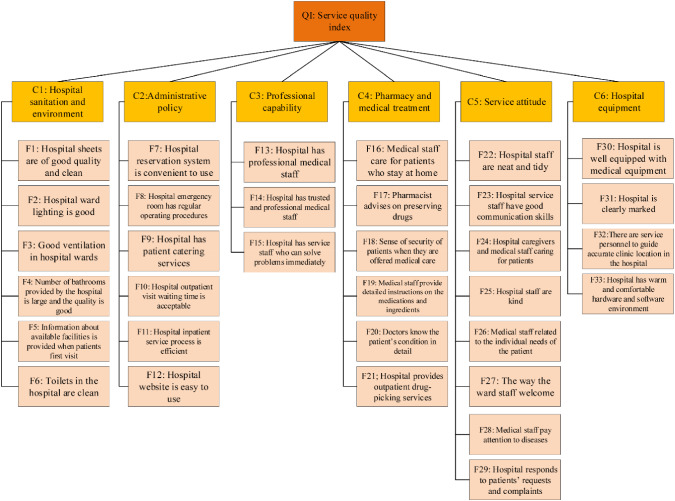
The structural criteria and sub-criteria to assess hospital service quality

Hospital service quality in a Metropolitan city is estimated by the proposed fuzzy Rough Copula Bayesian Network based on neighborhood operators’ decision-making approach. The health care service system has 200 beds capacity and 11 operation rooms. This hospital is allocated to the affected patients with COVID, with a high number of confirmed cases per 1 million people and considerable loss of medical service staff in the early stage of the SARS-CoV-2 outbreak. In addition, heavy daily patient circulation and an increasing number of confirmed severe cases requiring hospitalization are causing the hospital to face a lack of bed capacity. Increasing the workload of medical staff in a short period maximizes the need to sterilize the equipment and medical tools. Thus, this extensive workload is supposed to raise the number of confirmed cases and occupational accidents. As can be seen From Fig. 2, the alternatives and criteria of the present study to evaluate hospital service quality are obtained from [3, 5, 55].

The evaluation of hospital service quality includes 6 criteria (C1–C6) and 33 sub-criteria (F1–F33). Decision-makers knowledge and technical information are employed to establish the Bayesian Network model. The criteria and sub-criteria will cause the center of attention, which is called the service quality index (QI). The QI explains as a probabilistic service quality index, which indicates how much the understudy hospital good is in service quality in a range of zero and one. The QI can quantitively show the service quality of hospitals. Besides, for a single hospital, QI ranks the subfactors from the best to worth, and subsequently, corrective actions can be presented to improve the worth subfactors. To create the influence diagram (cause and effect), in the Bayesian Network of all 33 subfactors, 6 criteria and QI are named root nodes, intermediate nodes, and leaf nodes, respectively.

Using the input data obtained from 800 patients in a private hospital in Tehran Metropolitan, the size of the decision-making problem would be $33 \times 800$, which directly influences hospital service quality. In this problem, each problem has 800 data points to construct the marginal distribution that has been modeled in the Bayesian Network structure. The determined 33 variables obtained from the patients’ opinions act as evaluation indicators to evaluate the influence on the service quality index. It is also clear that the higher numerical value is showing much more promising with specific variables. Respecting the consistency of input data, all $33 \times 800$ collected from patients' and decision makers' opinions are normalized in intervals zero and one.

Concerning the different input data, #$C_{ 1} - \# C_{ 6}$ play the intermediates nodes in the influence diagram. Root cause analysis shows that the reason for intermediates nodes is based on the nodes #$F_{ 1} - \# F_{ 33}$. As a hierarchical structure, the nodes at different levels contribute to the node QI, which is located at the highest level. Obtaining the value of QI is the first task that can be defined as a functional node. QI describes the hospital quality index qualitatively. Using qualitative decision makers opinions based on a fuzzy Rough set, the functional node of QI is defined as the weighted sum of six criteria or intermediate nodes #$C_{ 1} - \# C_{ 6}$ following the Equation as $QI = 0.1C_{ 1} + 0.1C_{ 2} + 0.25C_{ 3} + 0.1C_{ 4} + 0.15C_{ 5} + 0.3C_{ 6}$.

An extension of fuzzy Rough set theory based on fuzzy $\beta$-neighborhood operators using Eq. (14) is utilized to illustrate the way of obtaining importance weight set, that is, {0.1, 0.1, 0.25, 0.1, 0.15, 0.3}. The type of data in the is most of the fuzzy Rough theory-based applications is IFNs (intuitionistic fuzzy numbers), obtained from the language terms’ translation. However, it is rare to derive IFNs from the current data for numerical data with ambiguity and uncertainty in practice. Therefore, it asked 800 patients to express their opinions on the more important criteria as an extra task. The 62 patients out of 800 share their judgment in qualitative terms. The collected qualitative terms. There are a couple of approaches such as that use proposed Pythagorean fuzzy numbers (PFNs) as a proper alternative for IFNs, such as in [56, 57]. Therefore, all collected input qualitative terms are transferred into the PFNs and then aggregated into a single PFN. Since all PFNs are obtained from every single criterion, using Eq. (14), the crisp importance weight for all criteria is then computed.

**Fig. 3:**
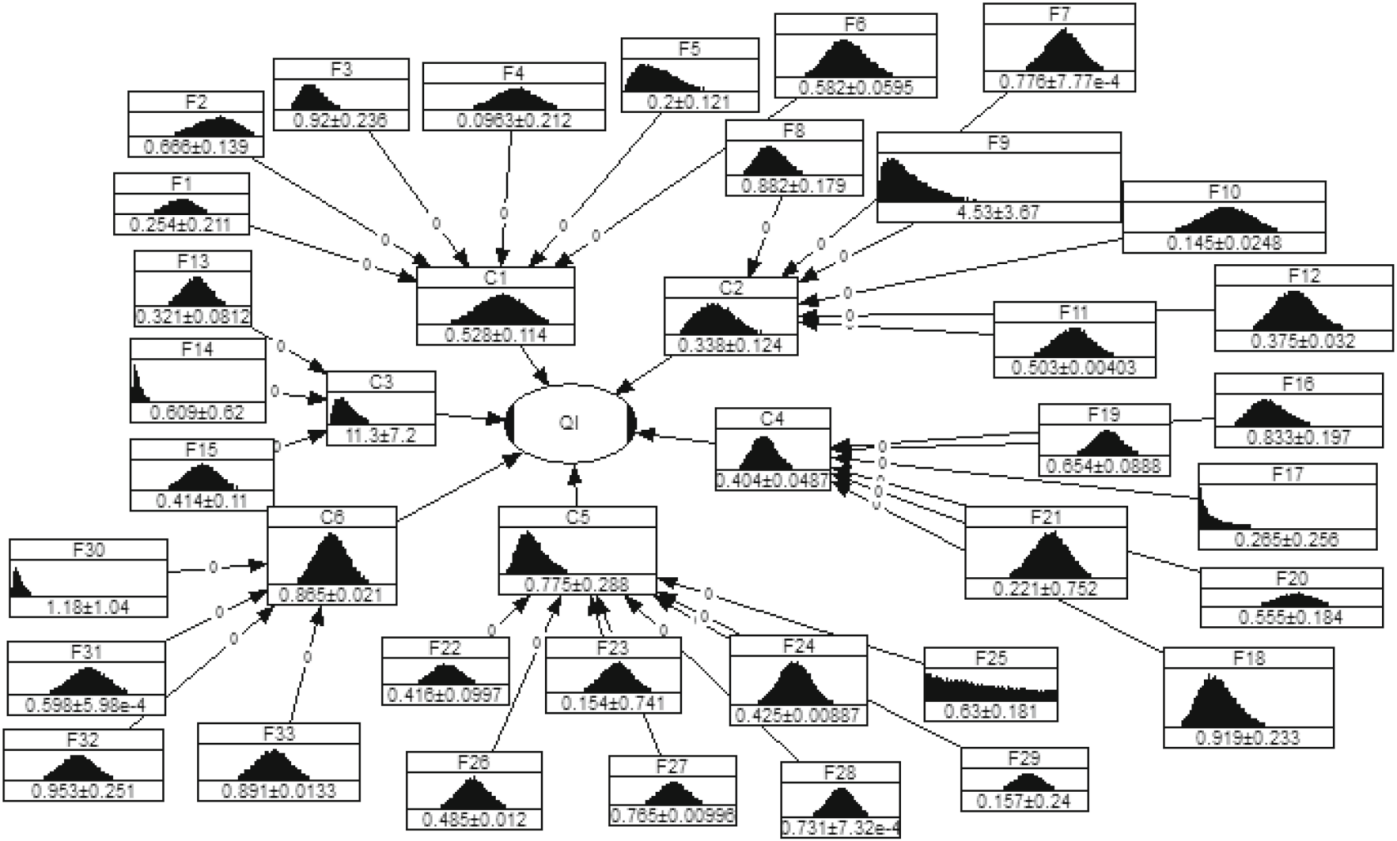
The Copula Bayesian Network evaluates the hospital's quality index

According to the QI function, criteria $C_{6}$ has a more significant impact on the result. Also, the linear function of QI shows the normalized evaluation of six intermediate nodes. As much as the #$C_{ 1} - \# C_{ 6}$ is close to the 1, which means they have better performance in Bayesian Network. However, the main important task is ranking the subfactors to find out that with the lowest rank and further corrective actions to be improved. Therefore, a group of decision makers identified the variables which they will thoroughly evaluate by Copula Bayesian Network. To model QI uncertainty in Bayesian Network, the main task is finding the proper marginal distribution for the continuous variables #$C_{ 1} - \# C_{ 6}$ and #$F_{ 1} - \# F_{ 33}$, indicating the corresponding probability distribution data learning. The marginal distributions can properly fit the extreme values compared to the empirical distributions. As listed in Table 2, three marginal distributions are engaged to model the empirical distributions of continuous variables #$C_{ 1} - \# C_{ 6}$ and #$F_{ 1} - \# F_{ 33}$ from learning data. Mainly, the six intermediate nodes #$C_{ 1} - \# C_{ 6}$ are somehow input variables, and the best marginal distributions would be fitted to their input data. The process of fitting marginal distribution for all 33 input variables. For every single variable, the AIC value of all three candidate distributions is compared to obtain the best-fitted marginal distributions considering the lowest AIC value. One of the main ways to obtain the marginal distributions is using the maximum likelihood approach. Table 3 provides the best-fitted marginal distribution for all input variables. These obtained marginal distributions should then be validated using Eq. (26). It is concluded that both marginal distributions and empirical data have compact shapes, which means that the marginal distributions have a high capability to be fitted to the empirical distributions. All marginal distributions are acceptable as they have a significant level of 0.5%, with $h = 0$ and $p$ value $> 0.05$. Accordingly, the bivariate Copula with consideration of interpretability and symmetry is integrated into the structured Bayesian Network model to characterize dependency between the variables-based Eq. (28), and therefore by computing the Euclidean distance between the empirical distributions (Eq. (27)) and another type of Copula function including t-Copula Gumbel Copula, and Frank Copula, the effectiveness of normal Copula can be verified. To show the dependency of the variables to reach the hospital service quality index (QI) with consideration of multivariate Copula relevant criteria and subfactors, the Gaussian Copula is used (Eqs. 23, 24). The corresponding Copula Bayesian Network is depicted in Fig. 3.

**Table 3:** The best-fitted marginal distributions for the input variables in Copula Bayesian Network

Input variables	Marginal distributions	Relevant parameters	Input variables	Marginal distributions	Relevant parameters
#$F_{ 1}$	Normal	$\mu = 0.254$, $\sigma = 0.211$	#$F_{ 21}$	Normal	$\mu = 0.221 , \sigma = 0.75$
#$F_{ 2}$	Beta	$\alpha = 7, \beta = 3.51 , a = 0, b = 1$	#$F_{ 22}$	Normal	$\mu = 0.416 , \sigma = 0.100$
#$F_{ 3}$	Log-normal	$\mu = - 0.114 , \sigma = 0.251$	#$F_{ 23}$	Normal	$\mu = 0.156 , \sigma = 0.741$
#$F_{ 4}$	Normal	$\mu = 0.098$, $\sigma = 0.156$	#$F_{ 24}$	Log-normal	$\mu = - 0.856 , \sigma = 0.021$
#$F_{ 5}$	Beta	$\alpha = 1.984 ,\beta = 7.895 , a = 0, b = 1$	#$F_{ 25}$	Log-uniform	$\mu = 0.156 , \sigma = 0.245 ,a = 0, b = 1$
#$F_{ 6}$	Log-normal	$\mu = - 0.547 , \sigma = 0.102$	#$F_{ 26}$	Normal	$\mu = 0.485 , \sigma = 0.012$
#$F_{ 7}$	Log-normal	$\mu = - 0.254 , \sigma = 0.001$	#$F_{ 27}$	Normal	$\mu = 0.756 , \sigma = 0.010$
#$F_{ 8}$	Log-normal	$\mu = - 0.145 , \sigma = 0.201$	#$F_{ 28}$	Log-normal	$\mu = - 0.313 , \sigma = 0.001$
#$F_{ 9}$	Gamma	$\alpha = 3, \beta = 1.5 , a = 0, b = 1$	#$F_{ 29}$	Normal	$\mu = 0.157 , \sigma = 0.24$
#$F_{ 10}$	Normal	$\mu = 0.145$, $\sigma = 0.025$	#$F_{ 30}$	Log-normal	$\mu = - 0.125 , \sigma = 0.754$
#$F_{ 11}$	Log-normal	$\mu = - 0.688 , \sigma = 0.008$	#$F_{ 31}$	Log-normal	$\mu = - 0.515 , \sigma = 0.001$
#$F_{ 12}$	Log-normal	$\mu = - 0.985 , \sigma = 0.085$	#$F_{ 32}$	Normal	$\mu = 0.954 , \sigma = 0.250$
#$F_{ 13}$	Normal	$\mu = 0.321 , \sigma = 0.081$	#$F_{ 33}$	Log-normal	$\mu = - 0.115 , \sigma = 0.015$
#$F_{ 14}$	Log-normal	$\mu = - 0.851 , \sigma = 0.845$	#$C_{ 1}$	Beta	$\alpha = 9.521 ,\beta = 8.546 , a = 0 , b = 1$
#$F_{ 15}$	Normal	$\mu = 0.414 , \sigma = 0.110$	#$C_{ 2}$	Beta	$\alpha = 4.568 ,\beta = 8.964 , a = 0 , b = 1$
#$F_{ 16}$	Log-normal	$\mu = - 0.212 , \sigma = 0.234$	#$C_{ 3}$	Gamma	$\alpha = 4.5 , \beta = 2.5 , a = 0 , b = 1$
#$F_{ 17}$	Log-uniform	$a = 0.25 , b = 1$	#$C_{ 4}$	Log-normal	$\mu = - 0.914 , \sigma = 0.121$
#$F_{ 18}$	Log-normal	$\mu = - 0.114 , \sigma = 0.251$	#$C_{ 5}$	Log-normal	$\mu = - 0.319 , \sigma = 0.361$
#$F_{ 19}$	Normal	$\mu = 0.654 , \sigma = 0.089$	#$C_{ 6}$	Normal	$\mu = 0.865 , \sigma = 0.021$
#$F_{ 20}$	Normal	$\mu = 0.556 , \sigma = 0.184$

To make a proper decision in assessing and evaluating the hospital quality service, correlation analysis, standard statistical analysis, and regression analysis is performed, in which the influence of each factor in the constructability of QI is assessed. Therefore, the factor with the highest correlation should be the lowest rank to receive corrective actions to improve the hospital service quality in the next assessment. The result of the studies above is provided in Table 4.

**Table 4:** The results of correlation analysis, standard statistical analysis, and regression analysis

Tag	Predicted variable	Names. base variable	E (predicted variable)	E (base variable)	Std (predicted variable)	Std (base variable)	Product moment correlation	Rank correlation	Regression coefficient	Correlation ratio	Linearity index	Partial correlation coefficient	Partial regression coefficient	Multiple correlation coefficient
1	QI	QI	3.328	3.328	1.788	1.788	1	1	1	1	0	1	− 1	1
2	QI	# C3	3.328	11.301	1.788	7.15	1	0.999	0.25	0.999	0	− 0.158	0.01	1
3	QI	# C5	3.328	0.776	1.788	0.289	0.025	0.025	0.155	0.001	0	− 0.133	0.005	1
4	QI	# F4	3.328	0.1	1.788	0.211	0.011	0.012	0.095	0	0	0	0	1
5	QI	# F27	3.328	0.765	1.788	0.01	0.011	0.011	1.919	0	0	0	0	1
6	QI	# C1	3.328	0.528	1.788	0.114	0.01	0.011	0.161	0	0	− 0.123	0.003	1
7	QI	# C4	3.328	0.404	1.788	0.049	0.003	0.007	0.125	0	0	− 0.088	0.002	1
8	QI	# F21	3.328	0.22	1.788	0.747	0.007	0.006	0.016	0	0	0	0	1
9	QI	# F32	3.328	0.954	1.788	0.25	0.005	0.006	0.033	0	0	0	0	1
10	QI	# F30	3.328	1.173	1.788	1.039	0.003	0.006	0.006	0	0	0	0	1
11	QI	# C2	3.328	0.337	1.788	0.123	0.004	0.006	0.059	0	0	− 0.125	0.003	1
12	QI	# C6	3.328	0.865	1.788	0.021	0.005	0.005	0.426	0	0	− 0.099	0.007	1
13	QI	# F12	3.328	0.375	1.788	0.032	0.006	0.005	0.33	0	0	0	0	1
14	QI	# F20	3.328	0.557	1.788	0.183	0.002	0.004	0.022	0	0	0	0	1
15	QI	# F6	3.328	0.582	1.788	0.06	0.003	0.004	0.095	0	0	0	0	1
16	QI	# F23	3.328	0.15	1.788	0.743	0.005	0.004	0.011	0	0	0	0	1
17	QI	# F10	3.328	0.145	1.788	0.025	0.002	0.003	0.129	0	0	0	0	1
18	QI	# F33	3.328	0.891	1.788	0.013	0.003	0.002	0.405	0	0	0	0	1
19	QI	# F11	3.328	0.503	1.788	0.004	− 0.002	0.002	− 0.765	0	0	0	0	1
20	QI	# F17	3.328	0.263	1.788	0.255	0	0.002	− 0.002	0	0	0	0	1
21	QI	# F31	3.328	0.598	1.788	0.001	0.001	0.001	1.608	0	0	0	0	1
22	QI	# F2	3.328	0.666	1.788	0.139	0.002	0.001	0.02	0	0	0	0	1
23	QI	# F25	3.328	0.631	1.788	0.181	0.001	0.001	0.005	0	0	0	0	1
24	QI	# F14	3.328	0.605	1.788	0.607	− 0.002	0.001	− 0.007	0	0	0	0	1
25	QI	# F18	3.328	0.92	1.788	0.236	0.002	0	0.019	0	0	0	0	1
26	QI	# F8	3.328	0.882	1.788	0.179	− 0.001	0	− 0.007	0	0	0	0	1
27	QI	# F5	3.328	0.201	1.788	0.122	0	− 0.001	− 0.004	0	0	0	0	1
28	QI	# F28	3.328	0.731	1.788	0.001	0.002	− 0.001	5.917	0	0	0	0	1
29	QI	# F7	3.328	0.776	1.788	0.001	− 0.004	− 0.002	− 9.494	0	0	0	0	1
30	QI	# F16	3.328	0.83	1.788	0.197	− 0.004	− 0.002	− 0.04	0	0	0	0	1
31	QI	# F19	3.328	0.655	1.788	0.089	− 0.004	− 0.003	− 0.074	0	0	0	0	1
32	QI	# F15	3.328	0.415	1.788	0.11	− 0.007	− 0.005	− 0.106	0	0	0	0	1
33	QI	# F1	3.328	0.254	1.788	0.211	− 0.004	− 0.005	− 0.032	0	0	0	0	1
34	QI	# F29	3.328	0.158	1.788	0.24	− 0.004	− 0.005	− 0.026	0	0	0	0	1
35	QI	# F22	3.328	0.416	1.788	0.101	− 0.007	− 0.005	− 0.125	0	0	0	0	1
36	QI	# F3	3.328	0.92	1.788	0.235	− 0.005	− 0.006	− 0.039	0	0	0	0	1
37	QI	# F26	3.328	0.485	1.788	0.012	− 0.006	− 0.006	− 0.912	0	0	0	0	1
38	QI	# F13	3.328	0.321	1.788	0.081	− 0.006	− 0.007	− 0.137	0	0	0	0	1
39	QI	# F9	3.328	4.501	1.788	3.688	− 0.007	− 0.008	− 0.004	0	0	0	0	1
40	QI	# F24	3.328	0.425	1.788	0.009	− 0.009	− 0.008	− 1.728	0	0	0	0	1

**Fig. 4:**
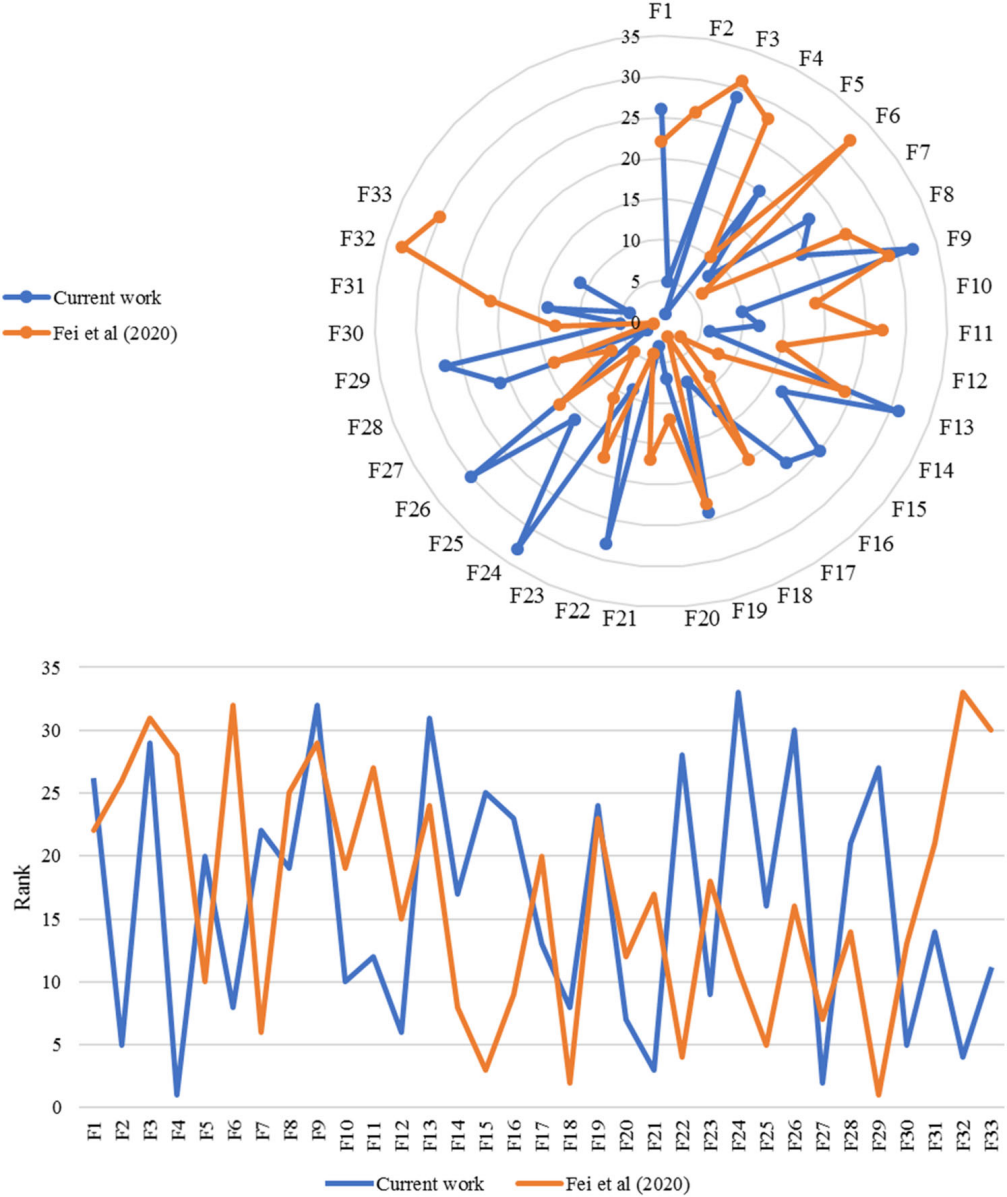
A comparison study based on the present study and in [3]

**Fig. 5:**
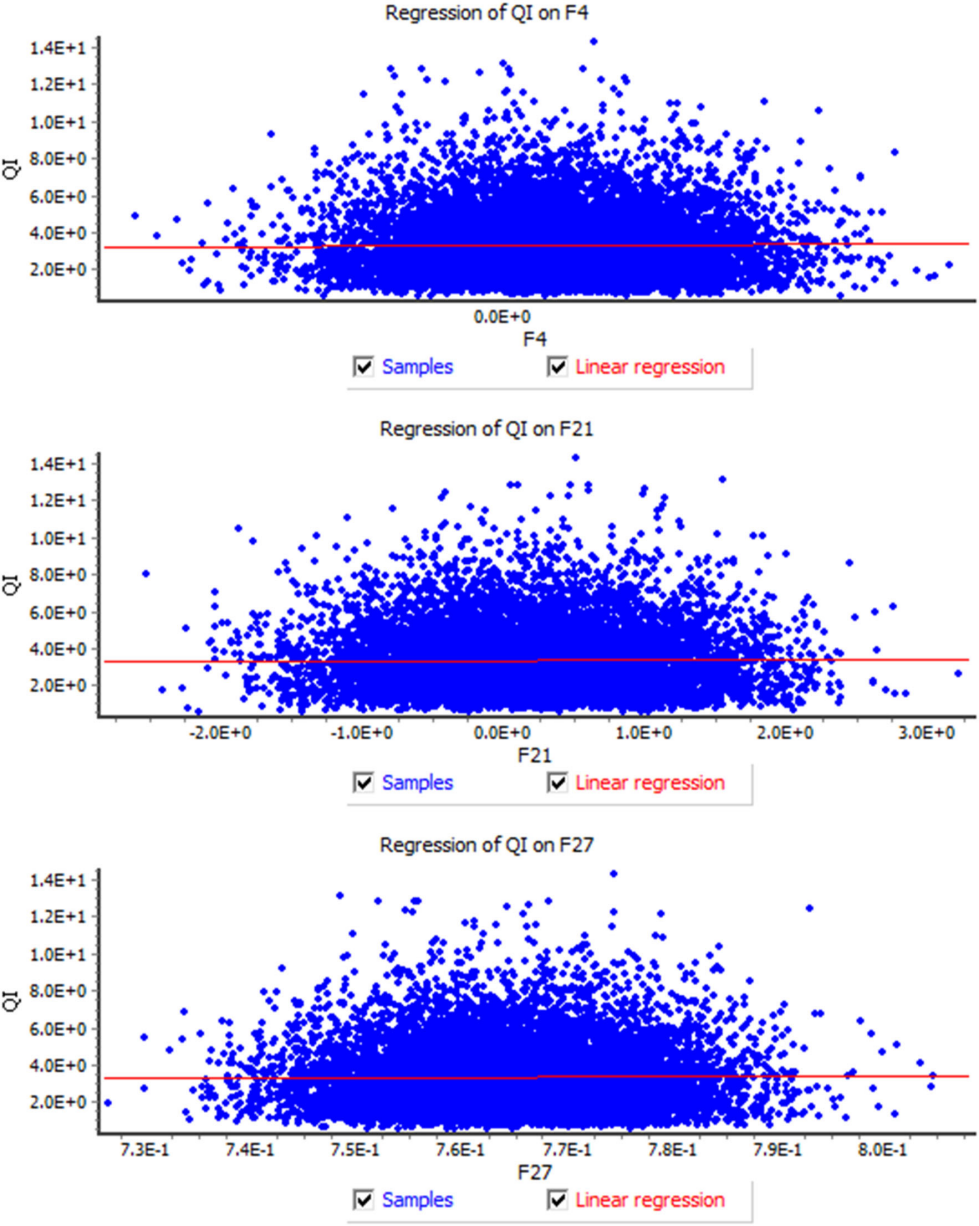
The correlation analysis between the most robust variables

As it can be seen from Table 4, subfactor #${F}_{ 24}$ (Hospital health caregivers and medical staff care for patients) has the lowest rank and needs to be improved by corrective actions. It is followed by #${F}_{ 9}$ (Hospital has patient catering services), #${F}_{ 13}$ (Hospital with professional medical staff), #${F}_{ 26}$ (Medical staff for individual requirements of the patient), and #${F}_{ 3}$(Good ventilation in hospital wards). A comparison of the results reached by the proposed approach and a novel BWM-based method with an extension of belief theory [3] is performed, see Fig. 4. It can be concluded that the two approaches present different results. However, this study has merits in consideration of multiple types of distributions, Log-Normal, Normal, and Beta, rather than only the Normal distribution considered in [3], and distributing weights to experts to avoid bias of experts’ opinions applied. Hence, the results computed by the proposed method tend to be more reliable and credible than those of other methods, such as in [3], and others that are disabled to consider the aforementioned aspects. The correlation analysis between the strongest variables is presented in Fig. 5 ((QI-#$F_{ 4}$(E(QI|#$F_{ 4} = 3.324 + 0.044{ }\# F_{ 4} - 0.137{ }\# F_{ 4}^{2} + 0.478{ }\# F_{ 4}^{3}$), QI-#$F_{ 21} ({\text{QI }}|{ }\# F_{ 21} = 3.317 + 0.008{ }\# F_{ 21} - 0.015{ }\# F_{ 21}^{2} + 0.001{ }\# F_{ 21}^{3} )$, and QI-#$F_{ 27} ({\text{QI }}|{ }\# F_{ 27} = - 456.007 + 1897.006 \# F_{ 27} - 2571.050{ }\# F_{ 27}^{2} + 1160.961{ }\# F_{ 27}^{3} )$), the sample is equal to 10,000).

**Fig. 6:**
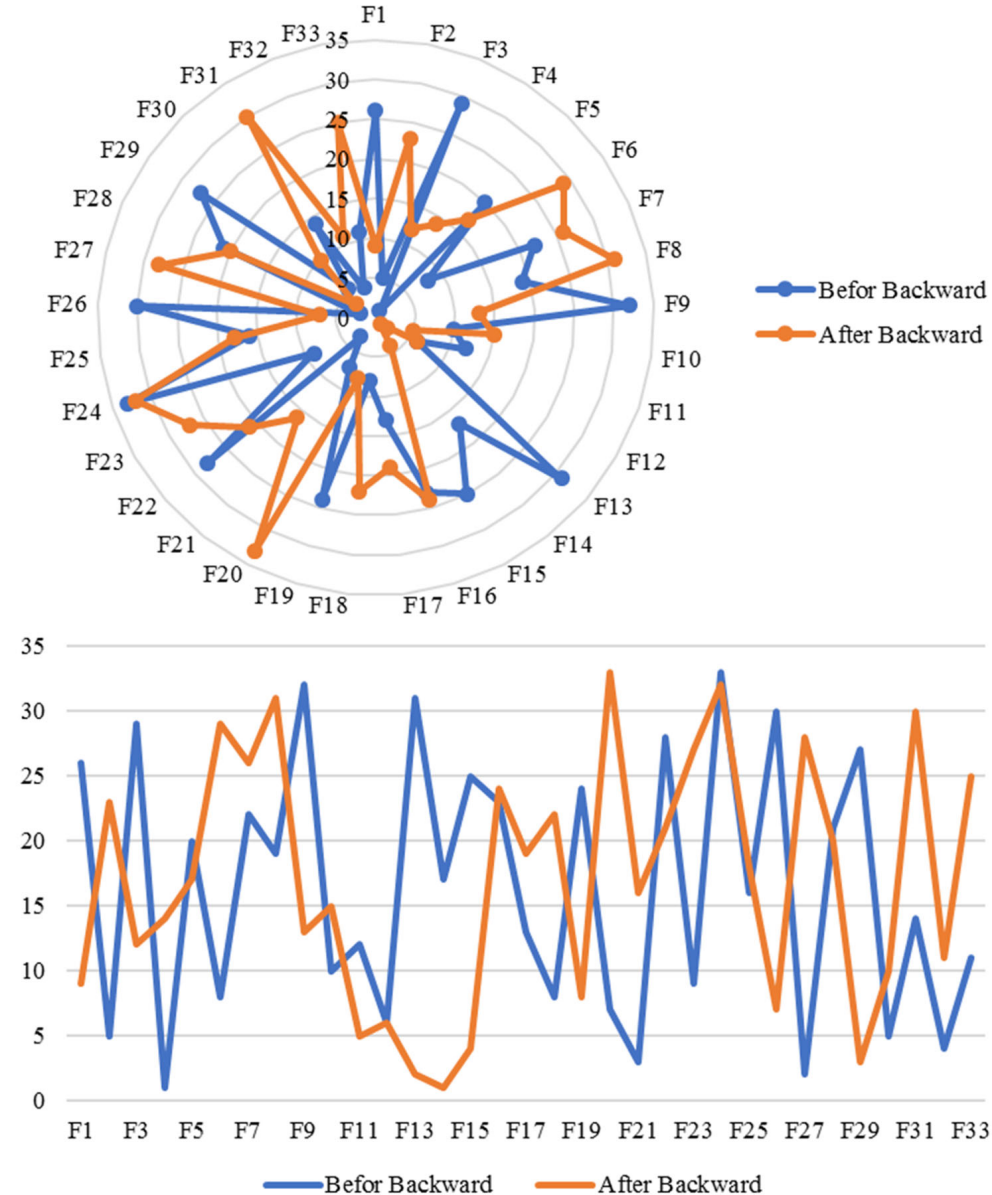
A comparison study before and after backward propagation

Another analysis is backward propagation using Eq. (32) is performed to obtain the optimal hospital quality index. As can be seen from Table 5, the results of the posterior probability of criteria and subfactors are provided. The correlation analysis between the most substantial variables is presented in Fig. 6. It can be understood that the optimal value of #$C_{ 1} - \# C_{ 6}$ and #$F_{ 1} - \# F_{ 33}$ to reach $QI = 1$ will change the priority of receiving corrective actions for the sub-actors to improve the hospital service quality for the next assessment turn.

**Table 5:** Backward propagation analysis of hospital service quality with posterior probability

Tag	Predicted variable	Names. base variable	*E* (predicted variable)	*E* (base variable)	Std (predicted variable)	Std (base variable)	Product moment correlation	Rank correlation	Regression coefficient	Correlation ratio	Linearity index	Partial correlation coefficient	Partial regression coefficient	Multiple correlation coefficient
1	QI	QI	1	1	0	0	1	1	0	1	0	− 1	0	0
2	QI	# F14	1	0.605	0	0.607	0	0.024	0	0	0	0	0	0
3	QI	# F13	1	0.321	0	0.081	0	0.023	0	0	0	0	0	0
4	QI	# F29	1	0.158	0	0.24	0	0.018	0	0	0	0	0	0
5	QI	# F15	1	0.415	0	0.11	0	0.017	0	0	0	0	0	0
6	QI	# F11	1	0.503	0	0.004	0	0.017	0	0	0	0	0	0
7	QI	# F12	1	0.375	0	0.032	0	0.016	0	0	0	0	0	0
8	QI	# C5	1	0.776	0	0.289	0	0.016	0	0	0	0	0	0
9	QI	# F26	1	0.485	0	0.012	0	0.015	0	0	0	0	0	0
10	QI	# F19	1	0.655	0	0.089	0	0.015	0	0	0	0	0	0
11	QI	# F1	1	0.254	0	0.211	0	0.015	0	0	0	0	0	0
12	QI	# C6	1	0.865	0	0.021	0	0.014	0	0	0	0	0	0
13	QI	# F30	1	1.173	0	1.039	0	0.014	0	0	0	0	0	0
14	QI	# C4	1	0.404	0	0.049	0	0.014	0	0	0	0	0	0
15	QI	# F32	1	0.954	0	0.25	0	0.013	0	0	0	0	0	0
16	QI	# F3	1	0.92	0	0.235	0	0.013	0	0	0	0	0	0
17	QI	# F9	1	4.501	0	3.688	0	0.013	0	0	0	0	0	0
18	QI	# F4	1	0.1	0	0.211	0	0.012	0	0	0	0	0	0
19	QI	# F10	1	0.145	0	0.025	0	0.012	0	0	0	0	0	0
20	QI	# F21	1	0.22	0	0.747	0	0.012	0	0	0	0	0	0
21	QI	# F5	1	0.201	0	0.122	0	0.012	0	0	0	0	0	0
22	QI	# F25	1	0.631	0	0.181	0	0.012	0	0	0	0	0	0
23	QI	# F17	1	0.263	0	0.255	0	0.012	0	0	0	0	0	0
24	QI	# F28	1	0.731	0	0.001	0	0.011	0	0	0	0	0	0
25	QI	# F22	1	0.416	0	0.101	0	0.011	0	0	0	0	0	0
26	QI	# F18	1	0.92	0	0.236	0	0.01	0	0	0	0	0	0
27	QI	# F2	1	0.666	0	0.139	0	0.01	0	0	0	0	0	0
28	QI	# F16	1	0.83	0	0.197	0	0.01	0	0	0	0	0	0
29	QI	# F33	1	0.891	0	0.013	0	0.01	0	0	0	0	0	0
30	QI	# F7	1	0.776	0	0.001	0	0.009	0	0	0	0	0	0
31	QI	# F23	1	0.15	0	0.743	0	0.007	0	0	0	0	0	0
32	QI	# F27	1	0.765	0	0.01	0	0.005	0	0	0	0	0	0
33	QI	# C2	1	0.337	0	0.123	0	0.005	0	0	0	0	0	0
34	QI	# F6	1	0.582	0	0.06	0	0.005	0	0	0	0	0	0
35	QI	# C1	1	0.528	0	0.114	0	0.005	0	0	0	0	0	0
36	QI	# C3	1	11.301	0	7.15	0	0.004	0	0	0	0	0	0
37	QI	# F31	1	0.598	0	0.001	0	0	0	0	0	0	0	0
38	QI	# F8	1	0.882	0	0.179	0	0	0	0	0	0	0	0
39	QI	# F24	1	0.425	0	0.009	0	− 0.001	0	0	0	0	0	0
40	QI	# F20	1	0.557	0	0.183	0	− 0.006	0	0	0	0	0	0

**Fig. 7:**
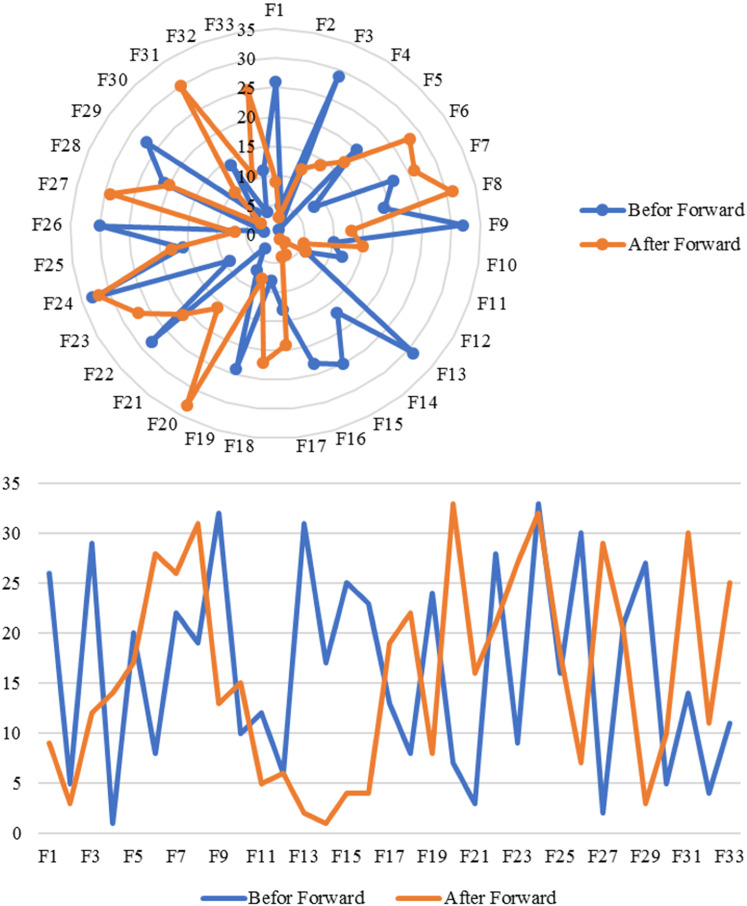
A comparison study before and after forward propagation based on the defined scenario

The last analysis is forward propagation or Copula Bayesian inference, which adds new evidence to the prior probability of variables. More specifically, the current version of the Network can continuously modify the newly added evidence(s). Therefore, the hospital quality index could be updated, subsequently, in the forward propagation using Eq. (27). It merely denotes that forward propagation is a supportive tool to update decision-making over time in different types of scenarios. In this study, we defined a scenario by changing the distributions of variables $\#{F}_{11},\#{F}_{18}$, and #${F}_{31}$ from lognormal into Beta distribution within parameters of $\alpha =8.5, 11.5, a=0, \mathrm{and} \, b=1$. The result of the forward propagation analysis is provided in Table 6. The correlation analysis between the strongest variables is presented in Fig. 7.

**Table 6:** Forward propagation analysis based on the defined scenario

Tag	Predicted variable	Names. base variable	*E* (predicted variable)	*E* (base variable)	Std (predicted variable)	Std (base variable)	Product moment correlation	Rank correlation	Regression coefficient	Correlation ratio	Linearity index	Partial correlation coefficient	Partial regression coefficient	Multiple correlation coefficient
1	QI	QI	1	1	0	0	1	1	0	1	0	− 1	0	0
2	QI	# F14	1	0.605	0	0.607	0	0.024	0	0	0	0	0	0
3	QI	# F13	1	0.321	0	0.081	0	0.023	0	0	0	0	0	0
4	QI	# F29	1	0.158	0	0.24	0	0.018	0	0	0	0	0	0
5	QI	# F15	1	0.415	0	0.11	0	0.017	0	0	0	0	0	0
6	QI	# F11	1	0.425	0	0.108	0	0.017	0	0	0	0	0	0
7	QI	# F12	1	0.375	0	0.032	0	0.016	0	0	0	0	0	0
8	QI	# C5	1	0.776	0	0.289	0	0.016	0	0	0	0	0	0
9	QI	# F26	1	0.485	0	0.012	0	0.015	0	0	0	0	0	0
10	QI	# F19	1	0.655	0	0.089	0	0.015	0	0	0	0	0	0
11	QI	# F1	1	0.254	0	0.211	0	0.015	0	0	0	0	0	0
12	QI	# C6	1	0.865	0	0.021	0	0.014	0	0	0	0	0	0
13	QI	# F30	1	1.173	0	1.039	0	0.014	0	0	0	0	0	0
14	QI	# C4	1	0.404	0	0.049	0	0.014	0	0	0	0	0	0
15	QI	# F32	1	0.954	0	0.25	0	0.013	0	0	0	0	0	0
16	QI	# F3	1	0.92	0	0.235	0	0.013	0	0	0	0	0	0
17	QI	# F9	1	4.501	0	3.688	0	0.013	0	0	0	0	0	0
18	QI	# F4	1	0.1	0	0.211	0	0.012	0	0	0	0	0	0
19	QI	# F10	1	0.145	0	0.025	0	0.012	0	0	0	0	0	0
20	QI	# F21	1	0.22	0	0.747	0	0.012	0	0	0	0	0	0
21	QI	# F5	1	0.201	0	0.122	0	0.012	0	0	0	0	0	0
22	QI	# F25	1	0.631	0	0.181	0	0.012	0	0	0	0	0	0
23	QI	# F17	1	0.263	0	0.255	0	0.012	0	0	0	0	0	0
24	QI	# F28	1	0.731	0	0.001	0	0.011	0	0	0	0	0	0
25	QI	# F22	1	0.416	0	0.101	0	0.011	0	0	0	0	0	0
26	QI	# F18	1	0.425	0	0.108	0	0.01	0	0	0	0	0	0
27	QI	# F2	1	0.666	0	0.139	0	0.01	0	0	0	0	0	0
28	QI	# F16	1	0.83	0	0.197	0	0.01	0	0	0	0	0	0
29	QI	# F33	1	0.891	0	0.013	0	0.01	0	0	0	0	0	0
30	QI	# F7	1	0.776	0	0.001	0	0.009	0	0	0	0	0	0
31	QI	# F23	1	0.15	0	0.743	0	0.007	0	0	0	0	0	0
32	QI	# F6	1	0.582	0	0.06	0	0.005	0	0	0	0	0	0
33	QI	# F27	1	0.765	0	0.01	0	0.005	0	0	0	0	0	0
34	QI	# C2	1	0.337	0	0.123	0	0.005	0	0	0	0	0	0
35	QI	# C1	1	0.528	0	0.114	0	0.005	0	0	0	0	0	0
36	QI	# C3	1	11.301	0	7.15	0	0.004	0	0	0	0	0	0
37	QI	# F31	1	0.425	0	0.108	0	0	0	0	0	0	0	0
38	QI	# F8	1	0.882	0	0.179	0	0	0	0	0	0	0	0
39	QI	# F24	1	0.425	0	0.009	0	− 0.001	0	0	0	0	0	0
40	QI	# F20	1	0.557	0	0.183	0	− 0.006	0	0	0	0	0	0

According to the analysis that has been performed, decision makers can obtain which factors have the lowest rank and need corrective actions to be received. Moreover, it can be understood what the outstanding value of each factor is to reach the optimum service quality index and how the model can be updated and be dynamic over time. Besides, in the case of a couple of hospitals, they can be compared together based on the value obtained from the service quality index for every single hospital. This may also affect receiving the budget, award, and system reputation.

### Comparison analysis

**Fig. 8:**
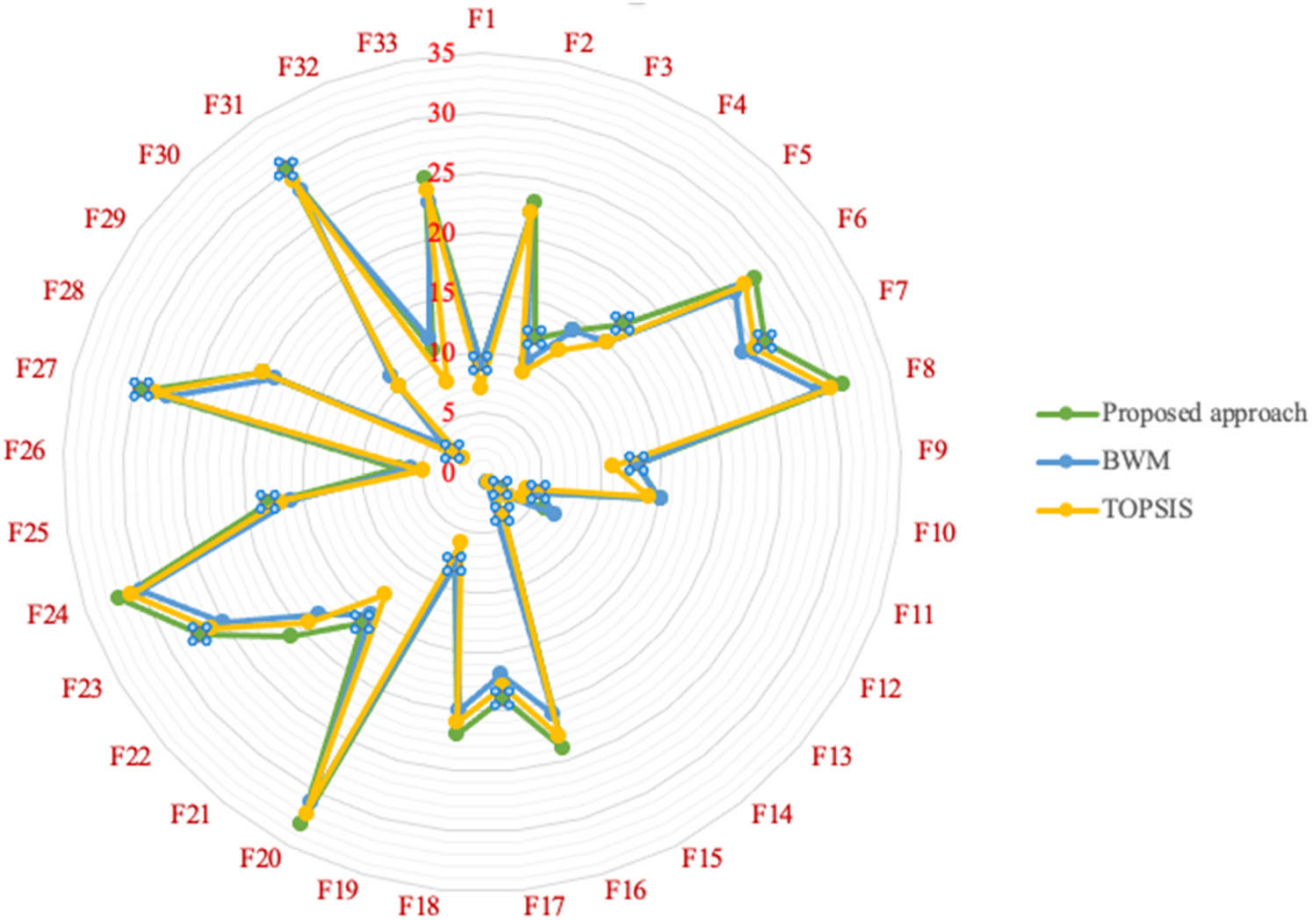
The comparison of the proposed approach with BWM, and TOPSIS

This subsection aims to determine the proposed methodology's feasibility and practicality via comparison analysis with initially two standard MCDM methods, and then with regular BN. In this subsection, the result of the proposed approach is compared with three different methods, including BWM [58], TOPSIS [59], and regular BN. The comparison outcomes among the proposed approach, BWM, and TOPSIS are presented in Table 7, illustrated in Fig. 8. This reflects that the priority of all solutions is entirely consistent with the first highest of the solutions. It simply means that the first solution in all methods is the same. This shows that the decision makers, based on some realistic restrictions such as time and complexity, can also rely on other types of methods. However, as reflected by the results of the two selected other models, this deduction is not valid for selecting the optimal solution.

**Table 7:** The factors and their corresponding priority ranking

Failure modes	Ranking		
	Proposed approach	BWM	TOPSIS
F1	9	9	7
F2	23	22	22
F3	12	10	9
F4	14	14	12
F5	17	15	15
F6	28	26	27
F7	26	24	25
F8	31	29	30
F9	13	13	11
F10	15	15	14
F11	5	5	4
F12	6	7	4
F13	2	3	3
F14	1	2	1
F15	4	1	4
F16	24	21	23
F17	19	17	18
F18	22	20	21
F19	8	8	6
F20	33	31	32
F21	16	15	13
F22	21	18	19
F23	27	25	26
F24	32	30	31
F25	18	16	17
F26	7	6	5
F27	29	27	28
F28	20	19	20
F29	3	4	2
F30	10	11	10
F31	30	28	29
F32	11	12	8
F33	25	23	24

In addition, the Spearman rank correlation coefficient is computed between each pair of methods, displayed in Table 8 to accurately reflect the conformity of the importance ranking of methods. Clearly, the greater Spearman correlation coefficient simply means higher conformity between the ranking techniques. As presented in Table 8, the ranking conformity of the proposed approach with other methods, BWM and TOPSIS, is greater than the ranking conformity with the rest of the pairwise comparisons. The conformity of failure modes priorities in comparison to the proposed approach with three other methods proves that the developed approach works correctly in the same direction as the other three methods, while the slight differences affirm the excellence proposed approach method due to its more robust mathematical structure versus the other methods. As mentioned in the methodology section, a physical explanation for this is that the proposed approach considers the different types of uncertainty, including process, model, subjective and objective input data.

**Table 8:** Spearman correlation coefficient of priority sequence among each pair of the proposed approach with BWM, and TOPSIS

Importance weight	Pairwise comparison		Spearman correlation coefficient
3	Proposed approach	TOPSIS	0.9956792
2	Proposed approach	BWM	0.9941180
1	BWM	TOPSIS	0.9900797

**Fig. 9:**
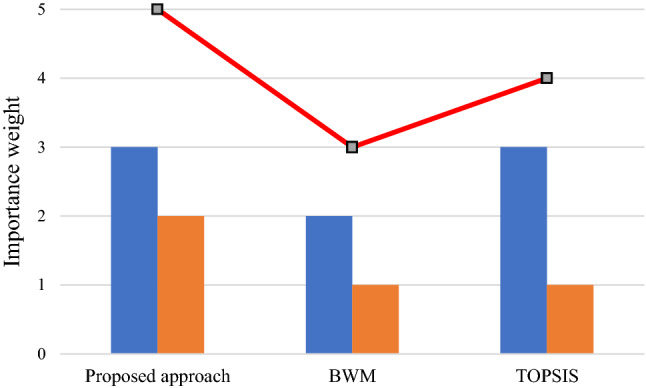
The importance ranking of among each pair of the proposed approach with BWM, and TOPSIS

According to the Spearman correlation coefficient, the importance weighting in descending order is provided, and the total ranking of the proposed approach with BWM and TOPSIS is depicted in Fig. 9. Thus, compared with BWM and TOPSIS, the proposed approach in this study is much more reliable and applicable in identifying the inter-relationship between different factors.

**Fig. 10:**
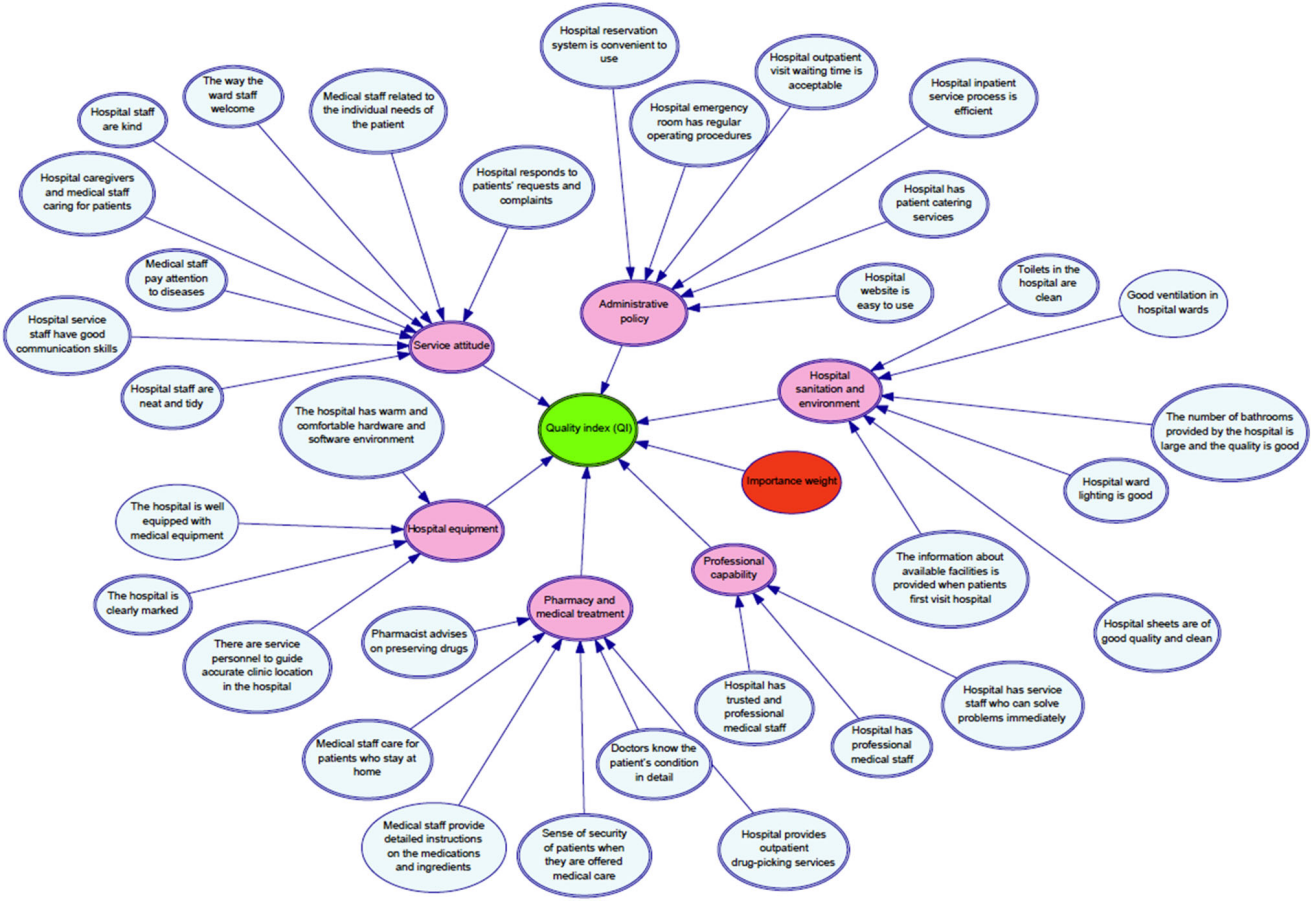
An illustration of regular BN is developed using GeNIe Modeler software

In the next comparison analysis, we developed regular BN considering the same input data and compared the outcomes with the proposed Fuzzy Rough Copula Bayesian Network model. As it can be seen from Fig. 10, an illustration of regular BN is developed using the GeNIe Modeler software package (https://www.bayesfusion.com/genie/). It should be added that, in the previous study conducted by authors [60], the regular BN is applied to assess the assess the quality index of a medical service. The input information for relevant alternatives (child nodes in Bayesian Network) is obtained from objective and subjective data. As an example, for the node alternative “Hospital staff are neat and tidy”, the percentage of how much this sentence is correct is obtained. Subsequently, the best-fitted distraction derived is the normal distribution. This process is continued for all nodes to obtain the best-fitted disruptions based on objective data or subjective opinions from decision makers. For the node obtained objectively, “Hospital has a professional medical staff”. It should be added that more than 90% of the data points are less than 80%, and the data focus on average values. The criticality analysis is carried out in the regular BN model to show the priority of failure modes and their contributions to the quality index. As it can be seen from Table 9, the failure mode priority in the proposed approach and regular BN is different, and the fact is that the proposed approach considers both objective and subjective uncertainty while the regular BN does not. The Spearman rank correlation coefficient is derived as 0.804489, which is less than the Spearman rank correlation coefficient of BWM and TOPSIS. However, regular BN, due to its capability to be updated over time, has much more advantages compared to the common MCDM tools.

**Table 9:** The factors and their corresponding priority ranking

Failure modes	Ranking	
	Proposed approach	Regular BN
F1	9	8
F2	23	24
F3	12	1
F4	14	9
F5	17	16
F6	28	21
F7	26	23
F8	31	29
F9	13	12
F10	15	13
F11	5	15
F12	6	5
F13	2	3
F14	1	6
F15	4	7
F16	24	20
F17	19	18
F18	22	27
F19	8	10
F20	33	32
F21	16	17
F22	21	22
F23	27	26
F24	32	33
F25	18	19
F26	7	33
F27	29	28
F28	20	14
F29	3	4
F30	10	2
F31	30	30
F32	11	11
F33	25	25

## Conclusion

This study proposes integrating the Copula Bayesian Network and fuzzy Rough set theory to assess, evaluate, and manage hospital service quality under an uncertain environment. The hospital service quality evaluation problem has been investigated by different researchers and several integrated methods. The current study used the Copula Bayesian Network to analyze the service quality, where a novel extension of fuzzy Rough set theory based on neighborhood operators is employed to tackle the subjective uncertainty of the problem. The designed framework integrates the Copula Bayesian Network and extends fuzzy rough set theory could rank a group of alternatives considering different criteria. In the present work, it is derived that the ${F}_{ 24}$ (Hospital health caregivers and medical staff care for patients) have the lowest rank and need to be improved by corrective actions. It is followed by #${F}_{ 9}$ (Hospital has patient catering services), #${F}_{ 13}$ (Hospital with professional medical staff), #${F}_{ 26}$ (Medical staff for individual requirements of the patient), and #${F}_{ 3}$(Good ventilation in hospital wards).

Based on the results obtained from the proposed approach, the following merits and advantages compared to MCDM tools can be highlighted:

- Copula Bayesian Network model can provide a better understanding of the causalities and the features in a complex system like hospital quality service, which many factors play a role in this regard.
- Copula Bayesian Network can also serve as a more convicting decision-making tool under objective uncertainty using different distributions and performing inference analysis over time.
- Utilizing a novel extension of fuzzy Rough set theory as a powerful tool can properly deal with inaccuracy. The advantage is that this does not necessarily require any prior knowledge beyond the data set, and in some studies with a lack of data could be a reliable choice.
- Using a new neighborhood operator in the proposed extension of fuzzy rough set theory can adequately satisfy reflexivity.

However, during the study, a couple of challenges have arisen in this study, which need to be considered as a direction for future work. First, in this study, the Clayton Copula is not evaluated as a tool; therefore, this should be considered with the three other types of Copula functions. Secondly, in this study, a method is proposed based on dealing with a combination of subjective and objective uncertainties, that is, while a combination of them is under discussion in literature; thus, it would be better to propose a method much more objectively or subjectively. Finally, using a hybrid methodology has extensive advantages in dealing with a complex decision-making problem; however, in practice, as a limitation, it makes time-consuming and cannot be a proper tool in an emergency decision-making problem. Therefore, such hybrid approaches need to be coded as an application. As the future direction, the probability theory can be integrated into MCDM methods alongside fuzzy concepts. Moreover, evaluating the service quality of different departments in a hospital is a potential topic for further studies.

## Data Availability

All data generated or analysed during this study are included in this published article.
